# Production and Characterization of Hydrothermal Extracts of the Needles from Four Conifer Tree Species: Scots Pine, Norway Spruce, Common Juniper, and European Larch

**DOI:** 10.1021/acssuschemeng.2c06406

**Published:** 2023-01-18

**Authors:** Omolara O. Mofikoya, Eemeli Eronen, Marko Mäkinen, Janne Jänis

**Affiliations:** Department of Chemistry, University of Eastern Finland, P.O. Box 111, FI-80101 Joensuu, Finland

**Keywords:** conifer, needle, hydrothermal extraction, high-resolution mass spectrometry, FT-ICR

## Abstract

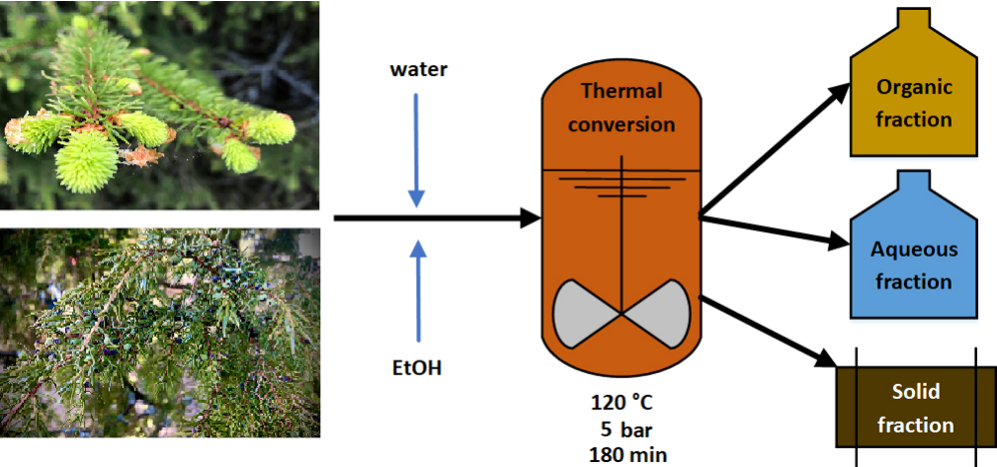

Coniferous trees are the most dominant trees in Finland with a great economic value for pulp, paper, and timber making. Thus, their utilization also results in large quantities of residues, especially bark and needles. Tree needles are a rich source of bioactive compounds, which have a considerable utilization potential in different pharmaceutical or techno-chemical applications. In this study, hydrothermal extraction (HTE) of the needles from four conifer tree species, namely, Scots pine, Norway spruce, common juniper, and European larch, was performed. Besides water, ethanol was also used as a solvent to enhance extraction efficiency and selectivity. All of the HTE experiments were conducted with a customized high-pressure reactor operated at 120 °C and 5 bar. The obtained needle extracts were then analyzed using a direct-infusion ultrahigh-resolution Fourier transform ion cyclotron (FT-ICR) mass spectrometry. The FT-ICR analysis of water and ethanol extracts allowed identification of over 200 secondary plant metabolites, including monosaccharides, organic acids, terpenoids, a variety of phenolic compounds, and nitrogen alkaloids. The use of ethanol as the extraction solvent considerably enhanced the recovery of lipids, especially terpenoids, some polyphenols, and other unsaturated hydrocarbon species.

## Abstract 2

Hydrothermal extraction is a promising technology for efficient and selective recovery of bioactive compounds from conifer tree needles.

## Introduction

Conifers are a group of cone-bearing gymnosperm plants that grow as trees or small shrubs, and they are found in most terrestrial habitats.^1^ They are also the dominant tree species in Finland, with Scots pine and Norway spruce being the most prevalent (67 and 22%, respectively), while European larch and common juniper make up only a small portion in the forested areas.^2^ Pine and spruce are of high economic value, being the major feedstocks for pulp, paper, and timber making. Apart from cellulose, hemicellulose, and lignin, conifers also contain a wide variety of extractives comprising volatile and nonvolatile compounds such as terpenoids and phenolic compounds. These secondary metabolites are accumulated in different parts of the trees, especially bark, roots, and needles, and they have an important role in the plants’ defense mechanism against biotic and abiotic stresses.^3^ They also possess considerable antibacterial, antifungal, and antioxidant activities.^3−6^ Such properties may find use in many pharmaceutical or techno-chemical applications. For instance, callus resin from spruce has been used in wound-healing resin salves, and some conifer terpenoids have shown positive therapeutic effects against anti-inflammatory diseases or cancer.

Several methods have been used to obtain extractives from conifer trees such as hot water extraction (HWE),^7^ simultaneous microwave–ultrasound-assisted extraction,^8^ or Soxhlet extraction.^9^ However, conventional extraction methods are time-consuming and often result in low yields. Over the past few years, there has been a steady development of new extraction methods such as supercritical fluid extraction (SFE), microwave-assisted extraction (MAE) or hydrothermal extraction (HTE).^10−13^ Different hydrothermal methods, such as HTE [also referred to as pressurized hot water extraction (PHWE), hydrothermal treatment (HTT), or subcritical water extraction (SWE)] have become more popular for the recovery of bioactive substances from different biomass feedstocks. HTE is a green extraction technique that uses water at an elevated temperature (typically 100–200 °C) and a moderate pressure (ca. 5–20 bar) to maintain water in its condensed state.^10^ In these conditions, the polarity (dielectric constant or relative permittivity) of water decreases considerably, and hydrogen bonds get weaker. Therefore, in these conditions, water behaves like many common organic solvents. There is also an increase in the mass transfer rates of compounds from the plant tissue matrix due to the decreased viscosity and lower surface tension of hot water.^14^ While most HTE experiments are performed in small-scale batch reactors, a pilot-scale pressurized hot water flow-through system has also been demonstrated.^15^ A thorough review on the application of hydrothermal methods to chemical conversion and transformation can be found elsewhere.^16^

Hydrothermal extraction has been efficiently utilized for the extraction of bioactive compounds from plant materials.^10,17−19^ The highest content of extractives in conifer trees is found in their foliage (needles), bark, knots, and stumps. Conifer needles may contain up to 40% of extractives by weight. There are only a few studies on the use of pressurized liquid extraction for the recovery of extractives from conifer bark, nuts, and seeds.^7,20^ However, no studies report on the use of hydrothermal methods for conifer needle extraction. In this work, hydrothermal extraction of the needles from four conifer species, namely, Scots pine, Norway spruce, common juniper, and European larch, was performed. Besides water, ethanol was also used as the extraction solvent to compare its extraction efficiency and selectivity toward certain compound classes. The obtained extracts were then characterized using a direct-infusion ultrahigh-resolution Fourier transform ion cyclotron resonance (FT-ICR) mass spectrometry, which allows for a rapid, nontargeted chemical fingerprinting of complex organic mixtures.

## Materials and Methods

### Plant Materials

The needles of Scots pine (*Pinus sylvestris*), Norway spruce (*Picea abies*), Common juniper (*Juniperus communis*), and European larch (*Larix decidua*) were collected between April and June from the Ylä-Valtimo region, North Karelia, Eastern Finland (63° 42′ N, 28° 52′ E), and stored in a cold room (4 °C) to avoid loss of the volatile components. The solvents used were HPLC grade water and ethanol obtained from VWR Chemicals (Darmstadt, Germany).

### Hydrothermal Extraction

Hydrothermal extraction experiments were carried out on a custom-built high-pressure reactor (Parr Series 4584 reactor: Parr Instrument Company Ltd, Moline, IL) with a reactor volume of 5.5 L and maximum operating temperature and pressure of 500 °C and 200 bar, respectively. For each extraction experiment, the reactor was loaded with 100 g of tree needles and 1000 g of water or (anhydrous) ethanol, after which the reactor was closed and pressurized to 5 bar with nitrogen gas. For efficient mixing, a flat blade stirrer was operated at 50 rpm for the whole duration of the process. The reactor was heated up to 120 °C using an automated heating program. The duration of the heating from the room temperature to 120 °C was approximately 60 min, and the reactor was kept at the target temperature for 60 min, after which it was cooled down within 60 min by the internal cooling coil, making the whole process time of about 180 min. The temperature trace obtained from the reactor thermocouple is shown in Figure S1. After the extraction process, the contents were removed from the reactor, followed by suction filtration to separate solid and liquid products. In this work, no post-reaction solvent removal by evaporation/distillation was performed to avoid the loss of the low-boiling-point components.

### Mass Spectrometry

All extracts were analyzed on a 12-T SolariX XR FT-ICR mass spectrometer (Bruker Daltonik, GmbH, Bremen, Germany) equipped with a dynamically harmonized ICR cell (ParaCell) and an Apollo-II ESI/APPI-II ion source. The samples were diluted 1:1000 (v/v) with methanol for the negative-ion ESI measurements and 1:100 (v/v) with methanol/toluene mixture (1:1 v/v) for the positive-ion APPI measurements. The samples were directly infused into the ion source with a syringe pump operated at a flow rate of 2 μL/min for ESI or 4 μL/min for APPI. Dry nitrogen was used as the drying (220 °C, 5 L/min) and nebulizing gas (0.8 bar). The mass calibration was done externally using sodium trifluoroacetate clusters or the APCI-L Tuning Mix (Agilent Technologies, Santa Clara, CA). The ions were detected at a *m*/*z* range of 98–1000 with a mass resolving power of ∼530,000 at *m*/*z* 300 (transient length 1.05 s). A total of 300 time-domain transients were co-added for each spectrum with a data size of 8 MWord. The data were processed in a magnitude mode with one zero-fill and full-sine apodization.

Before each sample run, a solvent blank was analyzed using the same parameters as with the samples to monitor any carryover effects. To avoid artifacts, the ions observed in the analytical blanks were compared to the sample measurements in both ionization modes.

Bruker ftmsControl 2.2 was used for the instrument control and data acquisition, while DataAnalysis 5.1 was used for the data post-processing and molecular formula assignments (SmartFormula tool). To improve mass accuracy, the mass spectra were further internally recalibrated using selected analyte ions. For the peak picking, the signal-to-noise (S/N) ratio was set to 5.0, and the relative threshold was 0.01%. For the molecular formula assignments, the parameters were as follows: elemental formula: ^12^C_1–100_^1^H_1–200_^14^N_0–2_^16^O_0–25_^32^S_0–1_; DBE ≤ 80; mass error ≤ 1 ppm. The initial structure annotations were accomplished using a Bruker CompoundCrawler database search engine. Microsoft Excel (Microsoft Corporation, Redmond, WA) and OriginPro 2018 (OriginLab Corporation, Northampton, MA) were used for data sorting and visualization.

### Compound Identifications

The detected compounds were tentatively identified by comparing the obtained molecular formulae to the compounds existing in several databases as well as those reported in earlier studies. The databases used were PubChem, KNApSAcK, Lipid Maps, Kegg, and Metabolomics Workbench. All compound identifications are considered either confidence level 3 (“tentative structure”) or 4 (“unique molecular formula”) identifications, as suggested by Schrimpe-Ruthledge et al.^21^ Level 3 identifications are typically obtained when accurate mass and isotopic pattern result in a limited number of candidate structures upon database search. In some cases, only a single reasonable structure matches with the obtained molecular formula, while in some other cases, a few isomeric structures may arise.

## Results and Discussion

### ESI/APPI FT-ICR MS Analysis of Conifer Extracts

To obtain detailed chemical compositions of the extracts, FT-ICR MS coupled with both (−) ESI and (+) APPI was used for the characterization. While ESI preferentially ionizes polar, oxygen-containing compounds (e.g., acids, phenols, and carbohydrates), APPI allows detection of less polar compounds (e.g., neutral lipids, phenolics, or unsaturated/aromatic hydrocarbons), thus providing complementary compositional information. One of the biggest advantages of FT-ICR MS is that it allows detection of thousands of compounds simultaneously without chromatographic separation. On the other hand, the biggest disadvantage of the technique is that the compounds having the same molecular formula (i.e., constitutional or stereoisomers) cannot be distinguished. After the deisotoping and clustering (e.g., removal of adducts), an average of 2,500 molecular features (i.e., unique isotopic compositions) were detected. The mass spectra obtained for each extract with (−) ESI and (+) APPI are shown in Figures S2 and S3. Most of the peaks spanned in the *m*/*z* range of 150–600.

### Visualization of the Overall Chemical Compositions of the Extracts

The chemical compositions of the extracts were compared using van Krevelen (VK) diagrams. The VK diagram is a scatter plot of the hydrogen-to-carbon (H/C) ratio as a function of the oxygen-to-carbon (O/C) ratio for each detected compound. The relative abundance of each analyte ion can be given by the size/color of the data point. The VK diagram is an effective means to visualize the overall chemical composition of a complex organic sample, as the compounds with similar elemental compositions (i.e., similar H/C and O/C ratios) are grouped and are present in different regions of the plot. The compounds were classified as (i) condensed aromatic structures (CAS), (ii) unsaturated hydrocarbons (UHC), (iii) phenolics, (iv) lipids, (v) amino acids (or other nitrogen-containing species), and (vi) carbohydrates, based on the regions they occupy in the VK diagram.

**Figure 1:**
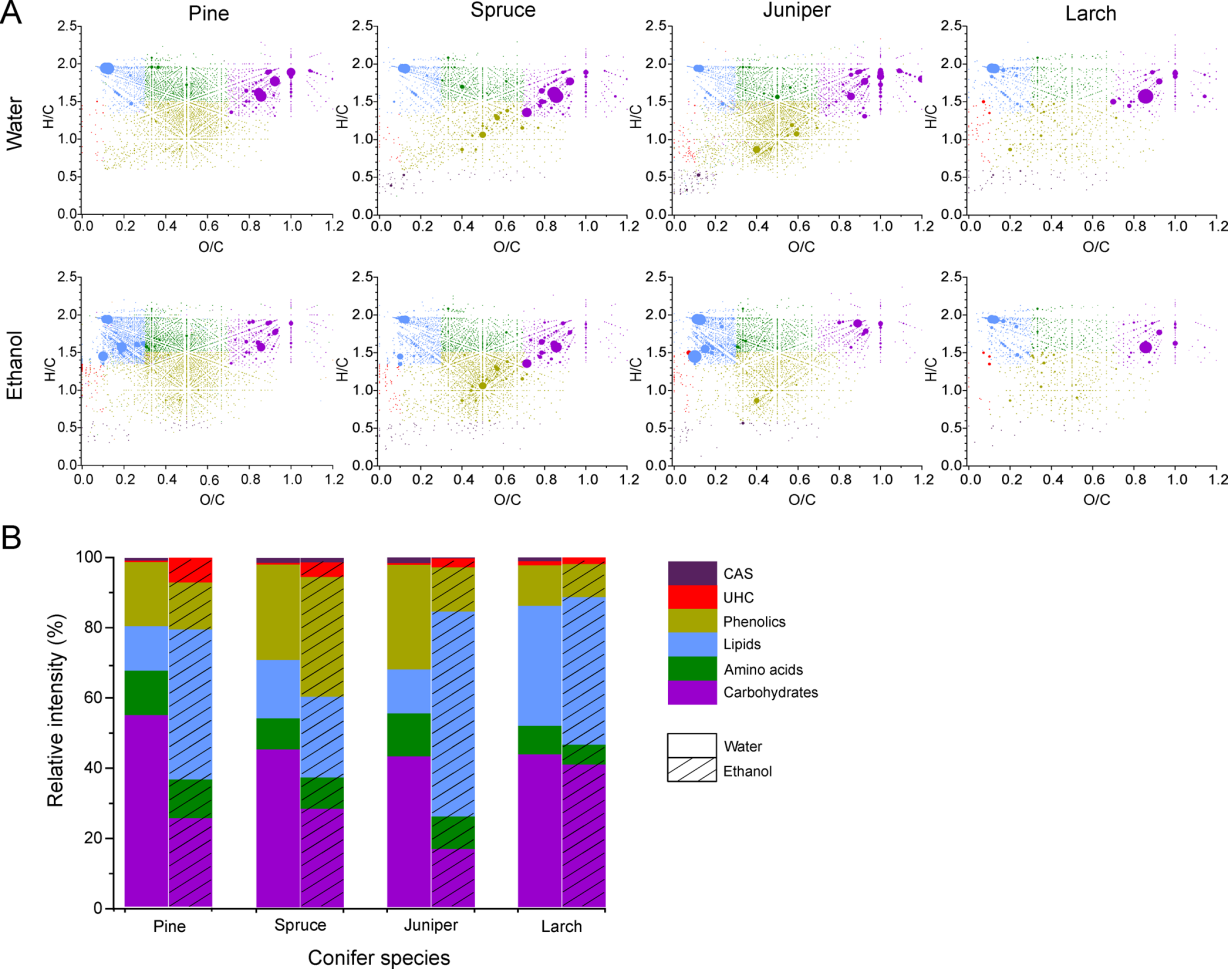
(A) Van Krevelen diagrams for the compounds detected in the needle extracts by (−) ESI FT-ICR MS. The dot color represents different compound classes as in panel (B), while the dot size represents the relative intensity. (B) Relative proportions of condensed aromatic structures (CAS), unsaturated hydrocarbons (UHC), phenolics, lipids, amino acids, and carbohydrates in water and ethanol extracts.

In the (−) ESI analysis (Figure 1a), highly abundant compounds were observed at H/C ≈ 1.5–2 and O/C ≈ 0.7–1, comprising a variety of organic acids as well as sugars and other polyols. The ethanol extracts had a few abundant species around H/C ≈ 1.5–2 and O/C ≈ 0.1–0.25, corresponding to a variety of lipids, especially saturated fatty acids and resin acids. To compare the chemical makeups of different extracts more quantitatively, the sum intensities of different compound classes were calculated and presented as stacked bar charts (Figure 1b). The most abundant classes were carbohydrates for the water extracts and lipids for the ethanol extracts, except for the spruce ethanol extract, which had the highest abundance of phenolics.

**Figure 2:**
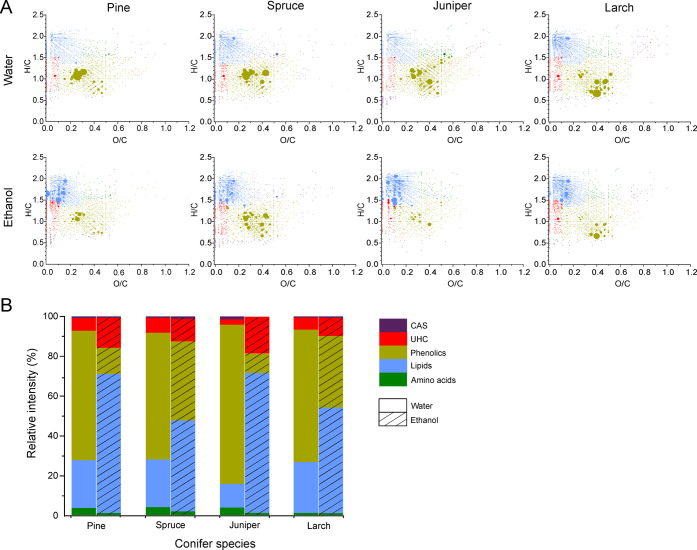
(A) Van Krevelen diagrams for the compounds detected in the needle extracts by (+) APPI FT-ICR MS. The dot color represents different compound classes as in (B), while the dot size represents the relative intensity. (B) Relative proportions of condensed aromatic structures (CAS), unsaturated hydrocarbons (UHC), phenolics, lipids, and amino acids in water and ethanol extracts.

In the (+) APPI analysis (Figure 2a), the most abundant species in the water extracts were found around H/C ≈ 0.8–1.2 and O/C ≈ 0.2–0.6, a typical region for phenolics. Furthermore, there were some species observed at high intensity in the region H/C ≈ 1–2 and O/C ≈ 0–0.5, corresponding to terpenoids (oxygenated terpenes), organic acids, or esters. In contrast, most ethanol extracts were enriched with lipids with a smaller relative proportion of phenolics. In addition, UHCs were more abundant in ethanol extracts. In general, UHC and CAS classes of compounds were the minor ones. Some terpene hydrocarbons (i.e., mono-, sesqui-, di-, and triterpenes) were also detected (H/C ≈ 1.4–1.6; O/C = 0), but their speciation is not possible without further chromatographic separation. Terpene hydrocarbons and terpenoids are the most important molecules for plants’ constitutive or induced defense mechanisms. Their profiles are influenced by developmental and environmental stimuli and can even vary among individual trees. No carbohydrates or other polyols were detected with (+) APPI. The “sugaric compounds” were effectively ionized by (−) ESI, while (+) APPI was more sensitive toward phenolics, lipids, and UHCs. The stacked bar charts showing the sum intensities for different compound classes are presented in Figure 2b.

### Compound Identifications

Direct-infusion FT-ICR MS is a powerful tool for the needs of plant metabolomics and lipidomics.^22^ Some compounds, such as fatty acids, can be unambiguously annotated solely based on the accurate mass data, but the other identifications are often regarded as “tentative identifications,”^23^ especially when multiple constitutional and/or stereoisomers occur. Tables S1 and S2 provide the lists of the tentatively identified compounds detected in the extracts. More than 200 compounds belonging to different chemical classes were identified.

### Carbohydrates

**Figure 3:**
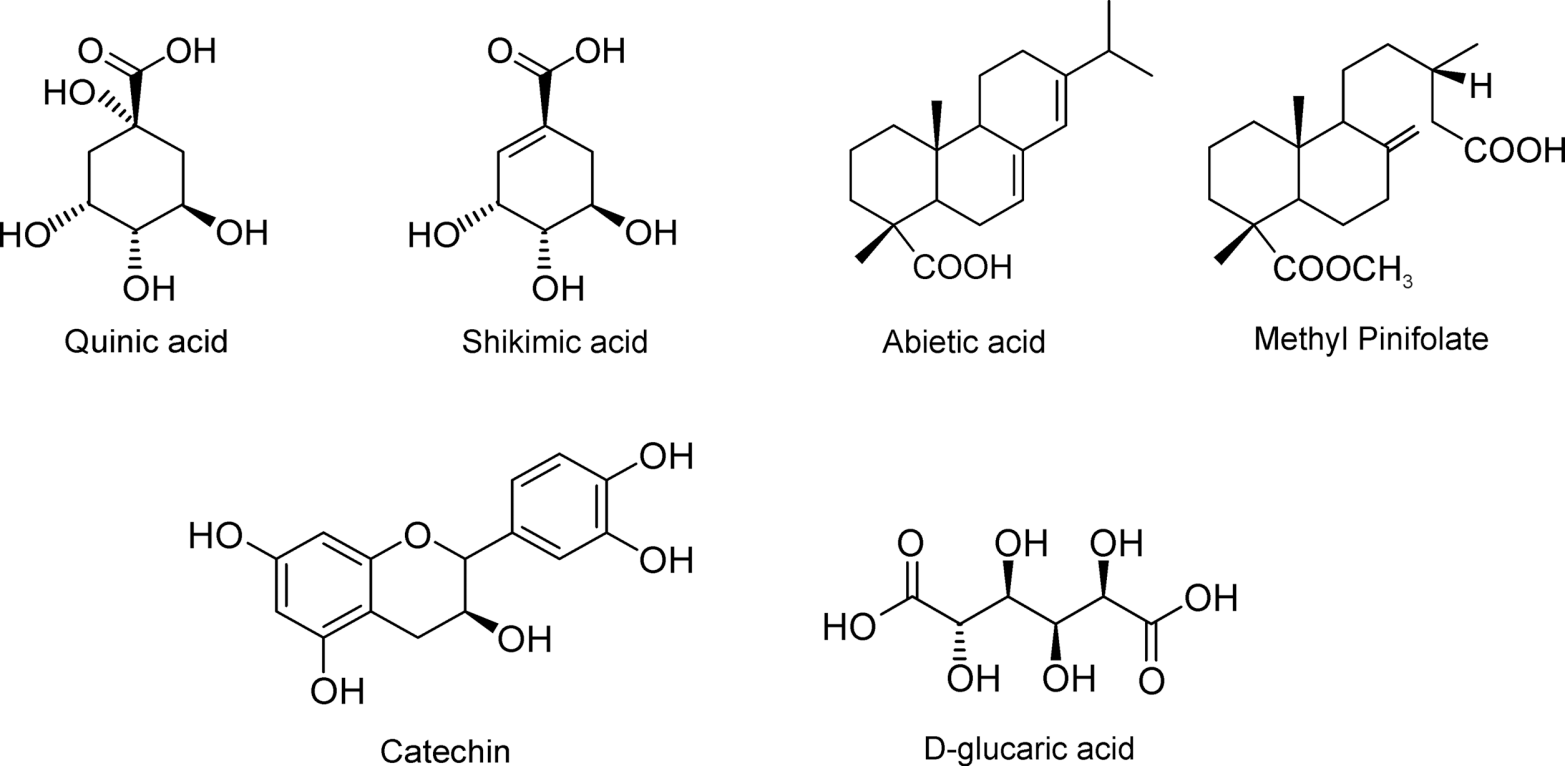
Structures of some abundant compounds detected.

Carbohydrates serve as the carbon energy sources for plant growth and are initially synthesized by a complex series of reactions via photosynthesis. Soluble carbohydrates have many physiological roles in tree growth, and their seasonal dynamics reflects different growth states, e.g., dormancy or new growth, as well as freeze protection during the cold season.^23^ The classes of compounds detected in the conifer extracts were different mono- and oligosaccharides, cyclitols, sugar acids, and uronic acids. Monosaccharides in the extracts were mostly represented by hexoses (C6), e.g., glucose. In addition, pentose (C5) monosaccharides (e.g., xylose) were quite abundant in the water extract and were also present in trace amounts in the ethanol extracts. A considerable amount of disaccharides (e.g., sucrose and rutinose) and their esters were also detected in the samples. Oligosaccharides were only found in minute quantities. Sugar acids, such as quinic acid, threonic acid, gluconic acid, glucaric acid, xylonic acid, and 4-*O*-methyl-α-d-glucuronic acid, were present in varying quantities. Quinic acid (Figure 3), which is a cyclitol, was the most abundant compound in the (−) ESI analysis of all extracts. In addition, galactosyl pinitol (a galactose cyclitol), which plays an important role in needle growth regulation, was detected as well. d-Glucaric acid (Figure 3), a precursor of glucuronic acid,^24^ was abundant in the larch extracts as compared to the other extracts. This agrees with the report by Dittrich et al. that D-glucaric acid occurs in large amounts in the needles of various *Larix* species.^24^ Glucuronic acid as well as 4-*O*-methyl-α-d-glucuronic acid have also been isolated from different parts of the conifer trees.^24−27^ Some sugar alcohols (e.g., arabitol and ribitol) were also detected.

### Lipids and Hydrocarbons

#### Fatty Acids

Saturated, long-chain fatty acids, ranging from nonanoic (C9:0) to behenic acid (C22:0), were detected in the extracts. Some unsaturated fatty acids were observed as well. In addition, a few hydroxy and dihydroxy fatty acids were also detected.

#### Tocopherols and Sterols

Tocopherols, a natural source of vitamin E, are present in almost all plant leaves, and they have been used as an indicator of enhanced oxidative stress in the needles of damaged trees.^28^ Tocopherols (α, β, δ, and γ) and tocotrienols (β, γ, and δ) were also detected in the extracts. They were effectively ionized by (+) APPI as compared to (−) ESI and were much more abundant in the ethanol extracts. α-Tocopherol was the main vitamin E isomer in all extracts. A profile of tocopherols found in *Pinus* species has been previously reported, and all of these compounds were found in this study.^29^ A phytosterol class was detected only by (+) APPI, β-sitosterol being the most abundant compound of this class. The other sterols present were campesterol, stigmasterol, and some triterpene sterols (e.g., cycloartenol and 24-methylenecycloartenol).

#### Resin Acids and Terpenoids

Terpenes represent the largest and most diverse class of plant secondary metabolites. They are present in almost every plant.^30^ Abietic acid and dehydroabietic acid are well-studied diterpenoids produced by conifers, together with a variety of mono- and sesquiterpenes, as the major component of resins. They are responsible for sealing of wounds, engulfing insects, and inhibiting potential pathogenic microorganisms in conifer trees.^31,32^ In both analyses, abietic acid was the most abundant diterpenoid in all of the ethanol extracts, except for pine, which had a diterpene hydrocarbon as the most abundant compound with (+) APPI and methyl pinifolate as the most abundant compound with (−) ESI. Methyl pinifolate (Figure 3), a methyl ester of pinifolic acid, was the most abundant compound in the pine extract, which agrees with our earlier study.^33^ Pinifolic acid was first reported in 1962 by Enzell and Theander, and it was shown to be highly enriched in pine needles (∼65% of the acid fraction of the acetone extract).^34^ A major portion of pinifolic acid has been reported to exist as its monomethyl ester.^35^ A free pinifolic acid was also detected at high abundance. Pinifolic acid and its methyl ester were only present in trace amounts in the other extracts and were completely absent in the spruce water extract. The other diterpenoids present were dehydroabietic acid, dehydropinifolic acid, anticopalic acid, ferruginol, dehydroferruginol, and 12-hydroxydehydroabietic acid. Monoterpenes and sesquiterpenes were dominant in the ethanol extracts, and traces were found also in the water extracts.

### Phenolics

#### Phenolic Acids

In this study, derivatives of hydroxycinnamic and hydroxybenzoic acids were tentatively identified. Hydroxycinnamic acids are the most widely distributed group of phenolic compounds.^36^ They can occur in their free forms or as the corresponding glycosides or combined with organic acids such as quinic acid.^36^ Cinnamic acid, ferulic acid, diferulic acid, coumaric acid, sinapinic acid, caffeic acid, and chlorogenic acid were detected in both water and ethanol extracts. 3-*p*-Coumaroylquinic acid was the most abundant hydroxycinnamic acid in both water and ethanol extracts of spruce but was present only in minute quantities in the other extracts. Free hydroxycinnamic acid was effectively ionized by (+) APPI while the combined form was detected by (−) ESI. Hydroxybenzoic acids, such as gallic acid, salicylic acid, and protocatechuic acid, were also tentatively identified.

#### Flavonoids

Flavonoids are present in a wide range of conifer species, and they are the most abundant phenolics in nature.^4^ Due to their structural diversity, they are further divided into different subclasses, i.e., anthocyanins, flavan-3-ols, flavones, flavanones, and flavonols. Flavonoids are aglycones in their basic structure, but a majority of them are present as glycosides in plants.^37^ The conifer extracts were concentrated with high amounts of flavonoids. Kaempferol was the most abundant flavonol present in the extracts, and it was quite dominant in the larch extract. Astragalin, a 3-*O*-glucoside of kaempferol, was present at moderate intensities in the (−) ESI analysis. Quercetin and its derivatives were found to be predominant, with the highest intensity in the juniper extracts. The compound observed at *m*/*z* 609.146406 with (−) ESI was tentatively identified as rutin and was quite abundant in the juniper water extract compared to the rest of the samples. This is in line with the previous study by Olech et al.^38^ The other flavonols present were isorhamnetin, myricetin, laricitrin, syringetin, and their derivatives.

Another subclass that was quite abundant is flavan-3-ols. Catechin was the most abundant flavan-3-ol observed in all of the extracts, having the highest intensity in both ESI and APPI analysis of the juniper extracts. Catechin has been reported as the most abundant flavonoid aglycone in juniper,^38^ having considerable antioxidant activity.^39^ It has been reported that the concentration of catechin is influenced by province or location of the tree.^40^ Catechin-3-glucoside was also detected in the juniper extracts but was present only in trace amounts in the other extracts. Another derivative of catechin present was gallocatechol, which was observed in high abundance.

Other flavonoids tentatively identified were aromadendrin, ampelopsin, and taxifolin (dihydroflavonols), narigenin (flavanone), and apigenin (flavone). They were present as free and their glucoside forms; the aglycones were detected by both (−) ESI and (+) APPI, and the corresponding glucosides were detected by (−) ESI. Bioactivity profiles of these compounds have been reported previously.^4,5,38^

#### Stilbenes

Stilbenes are natural defense polyphenols that are found in many plant species. They are one of the most abundant phenolic compounds found in spruce and pine trees.^5^ Stilbenes are the most studied compounds in the bark of Norway spruce, and they occur both as free aglycones and the corresponding glucosides.^41^ Stilbene glucosides, such as isorhapotin, astrigin, and piceid, have been reported as the major stilbenes in Norway spruce,^5,41^ and this was also true in the present study. Isorhapotin was the most abundant compound found in the spruce extract. Piceid was detected as the minor constituent in spruce, consistent with the results of Mulat et al.^41^ These compounds were present in trace amounts in the other extracts. The corresponding aglycones, isorhapontigenin, piceatannol, and resveratrol were also present in this work and were found at high intensity in the spruce extract. The antibacterial activities of these compounds have been reported.^5^

#### Lignans

Lignans are the dimers of coniferyl or sinapyl alcohols.^39^ They are responsible for defending plants against ultraviolet light, oxidative stress, and a variety of pathogens.^42^ Taxiresinol, lariciresinol, pinoresinol, secoisolariciresinol, and hydroxymataresinol are examples of lignans tentatively identified in the extracts. Several biological properties such as antioxidant, antitumor and antimicrobial activities have been reported for these compounds.^5,39,42^

#### Condensed Tannins

Condensed tannins, also called proanthocyanidins, are oligomers and polymers of flavan-3-ols. The condensed tannins detected in our samples were procyanidin A2, procyanidin B1, procyanidin trimer C1, prodelphinidin A, and prodelphinidin B. They were detected by both (−) ESI and (+) APPI. Procyanidins are polymers formed from catechin, while prodelphinidins are formed from gallocatechin units. Tannins can be used for tanning of leather due to their ability to make complexes with proteins, and they also possess antimicrobial properties.^5^

Other phenolic compounds tentatively identified were piceol (4-hydroxyacetophenone) and its glucoside, picein.

### Piperidine Alkaloids

Piperidine alkaloids are the minor secondary metabolites in the *Pinaceae* species, and they play a crucial role in the defensive chemistry of trees. The alkaloids detected in the present study were 1,6-hydropinidine and 1,6-dehydropinidinone. They were present only in the spruce water extract, which is in line with our previous study of spruce sprout chemistry.^43^ These compounds were detected only by the (+) APPI analysis.

### Other Compounds

The other major compounds identified were shikimic acid, a cyclitol that was quite abundant in the pine and spruce extracts. Other interesting organic compounds, such as vanillin, malic acid, and adipic acid, were identified as well. The volatile compounds, such as thymol, verbenene, isoeugenol, linalool oxide, limonene, and linalool, were tentatively identified.

## Conclusions

Conifer needles contain a vast amount of bioactive secondary metabolites, which may find use in pharmaceutical, nutraceutical, or techno-chemical applications. Efficient and selective recovery techniques are therefore essential to recover these substances from the needle materials. In this study, hydrothermal and solvothermal extracts were obtained from the conifer needles of four tree species, and their chemical fingerprints were determined using direct-infusion FT-ICR mass spectrometry coupled with ESI and APPI ionizations. The results obtained in this study indicate that HTE is an effective extraction method for obtaining bioactive compounds from conifer needles. The conditions used in the HTE experiments allowed efficient extraction of a wide range of compounds, both volatile and nonvolatile ones. By tailoring the extractant (e.g., water to ethanol), specific classes of compounds can be selectively recovered. After the extraction, the solvent used can be recycled by simple vacuum distillation. The direct-infusion ESI/APPI FT-ICR MS used in this study allowed a comprehensive analysis of hundreds of secondary metabolites in each sample, showing its superiority in complex organic mixture analysis.

## Supporting Information Available

Temperature profile for the hydrothermal extraction; (−) ESI/ (+)APPI mass spectra of the extracts; and compounds identified with (−) ESI/ (+)APPI from the conifer extracts (PDF)
