# Supplementary material for: Production and Characterization of Hydrothermal Extracts of the Needles from Four Conifer Tree Species: Scots Pine, Norway Spruce, Common Juniper, and European Larch
Source: ACS Sustain Chem Eng. 2023 Jan 18;11(4):1540–7. doi: 10.1021/acssuschemeng.2c06406 (PMC9891111; doi:10.1021/acssuschemeng.2c06406)
Supplement: Supplementary file 1 — sc2c06406_si_001.pdf [file sc2c06406_si_001.pdf]

Supporting Information

Production and Characterization of Hydrothermal Extracts of the Needles from Four Conifer Tree Species: Scots Pine, Norway Spruce, Common Juniper, and European Larch

*Omolara O. Mofikoya, Eemeli Eronen, Marko Mäkinen, and Janne Jänis**

University of Eastern Finland, Department of Chemistry, P.O. Box 111, FI-80101 Joensuu, Finland

**Corresponding author: Email: janne.janis@uef.fi, Tel. +358-50-4601057*

Number of Pages: 20

Number of Figures: 3

Number of Tables: 2

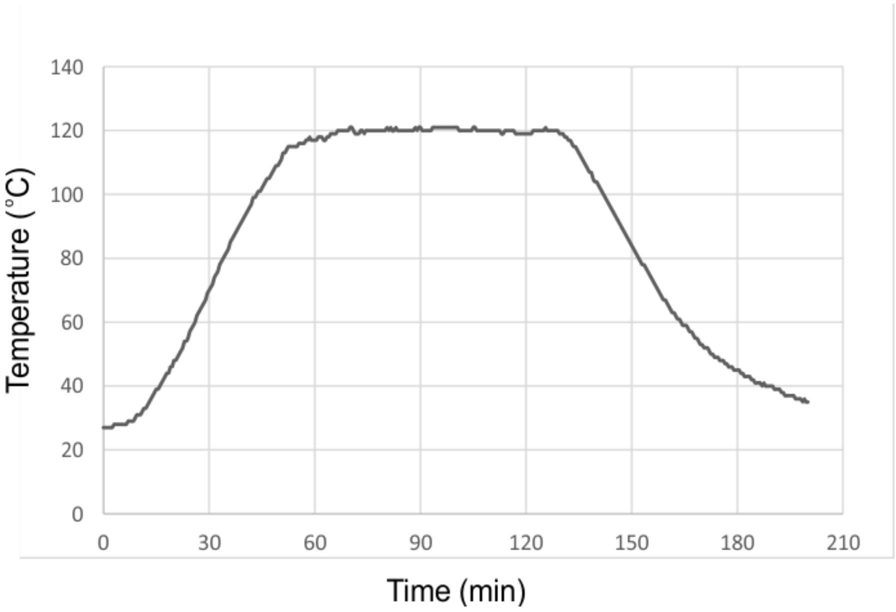

Figure S1. Temperature profile for the hydrothermal extraction.

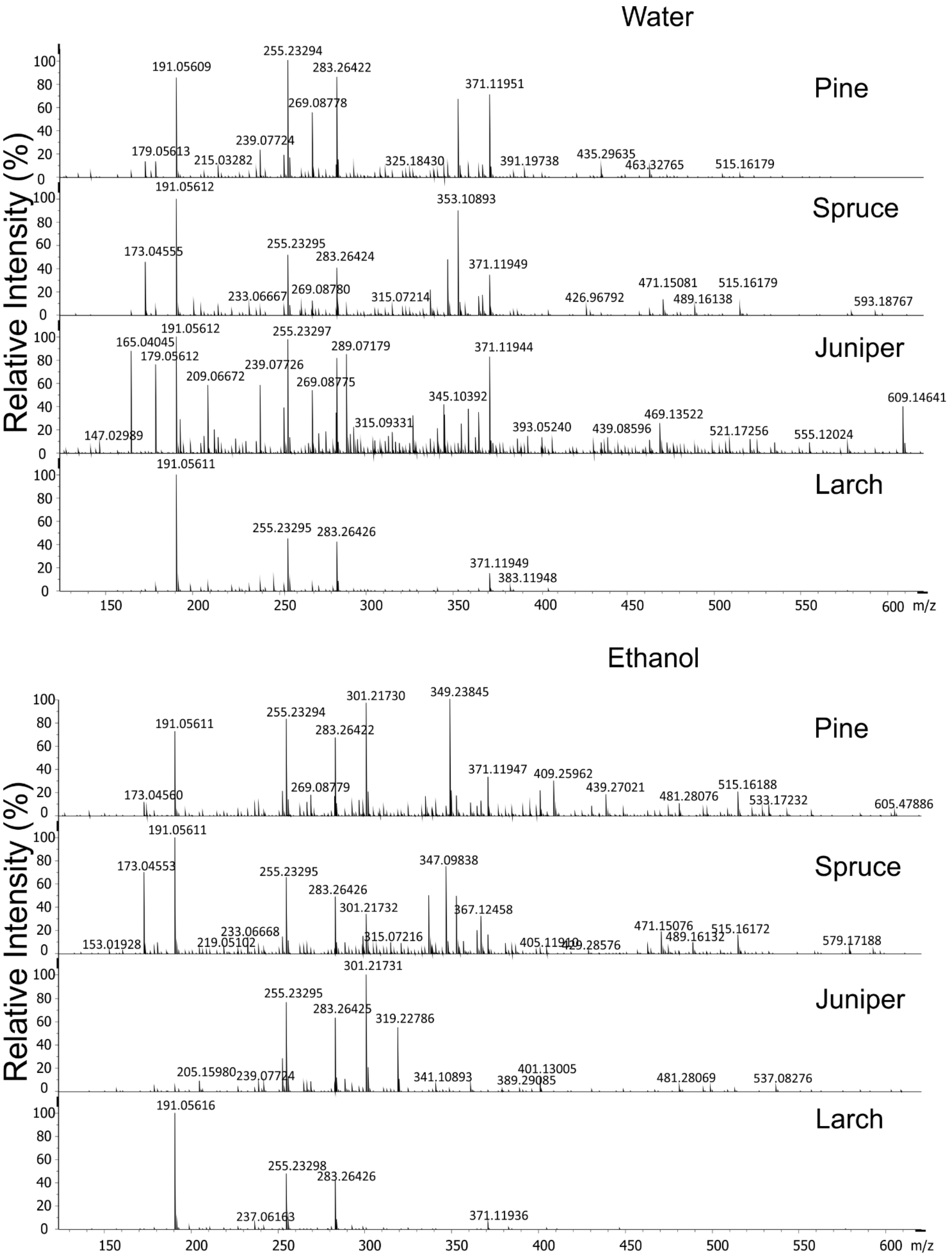

Figure S2. (–) ESI mass spectra of the extracts.

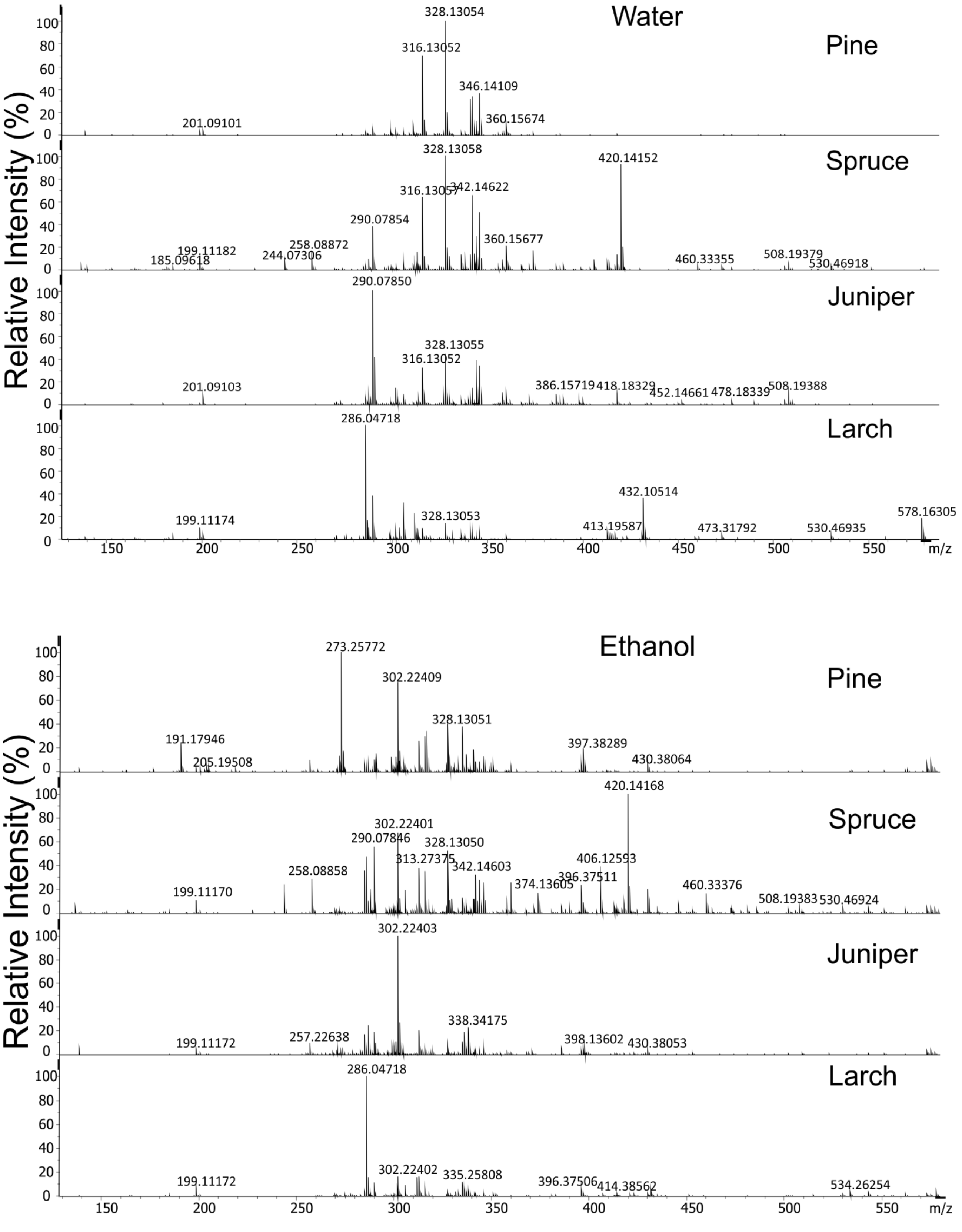

Figure S3. (+) APPI mass spectra of the extracts.

Table S1. Compounds identified with (–) ESI from the conifer extracts.

Calculated *m/z*	Observed *m/z*	Elemental formula	Error (ppm)	Relative Intensity (%)								Tentative compound(s)	Class
				Water				Ethanol					
				Pine	*Spruce*	*Juniper*	*Larch*	*Pine*	*Spruce*	*Juniper*	*Larch*		
105.019332	105.019330	$C_3H_6O_4$	0.023	N.A	0.0038	0.0056	N.A	0.0027	0.0132	N.A	N.A	Glyceric acid	Organic acid
115.003682	115.003691	$C_4H_4O_4$	-0.073	N.A	0.0065	0.0020	N.A	0.0041	0.0172	0.0077	N.A	Fumaric acid	Organic acid
117.019332	117.019335	$C_4H_6O_4$	-0.023	0.0140	0.0543	0.0208	0.0117	0.0226	0.0752	0.0243	0.0097	Succinic acid	Organic acid
121.029503	121.029509	$C_7H_6O_2$	-0.051	0.0122	0.0322	0.0089	0.0283	0.0176	0.0718	0.0246	0.0189	Benzoic acid	Organic acid
125.024418	125.024430	$C_6H_6O_3$	-0.096	0.0087	0.0050	0.0073	N.A	0.0096	0.0117	0.0081	N.A	Pyrogallol	Monophenol
133.014247	133.014247	$C_4H_6O_5$	-0.001	0.0315	0.2960	0.0740	0.0645	0.0417	0.1249	0.0162	0.0485	Malic acid	Organic acid
135.029897	135.029906	$C_4H_8O_5$	-0.063	0.0909	0.0597	0.1716	0.0146	0.0289	0.0394	0.0223	0.0158	Threonic acid	Organic acid
135.045153	135.045170	$C_8H_8O_2$	-0.126	N.A	0.0692	0.0044	N.A	0.0065	0.0958	0.0055	N.A	Piceol	Monophenol
137.024418	137.024413	$C_7H_6O_3$	0.033	0.0392	0.0736	0.0653	0.0229	0.0509	0.2464	0.0691	N.A	Salicyclic acid	Organic acid
147.045153	147.045141	$C_9H_8O_2$	0.081	N.A	0.0045	0.0056	N.A	N.A	0.0127	N.A	N.A	Cinnamic acid	Organic acid
149.045547	149.045550	$C_5H_{10}O_5$	-0.018	0.0696	0.0293	0.0871	0.0570	0.0112	0.0191	0.0187	0.0108	Xylose	Carbohydrate
151.040068	151.040052	$C_8H_8O_3$	0.107	0.0199	0.0314	0.0296	0.0142	0.0181	0.0626	0.0347	0.0143	Vanillin	Organic acid
151.061197	151.061179	$C_5H_{12}O_5$	0.122	0.0163	0.0081	0.0179	0.0667	0.0147	0.0151	0.0325	0.0319	Arabitol, Ribitol	Carbohydrate
153.019332	153.019316	$C_7H_6O_4$	0.108	0.0918	0.0537	0.0923	0.0419	0.1985	0.6227	0.1711	0.0608	Protocatechuic acid	Phenolic acid
161.045547	161.045537	$C_6H_{10}O_5$	0.062	0.1086	0.0506	0.0717	0.0948	0.1593	0.4159	0.2941	0.1116	Levoglucosan	Carbohydrate
163.040068	163.040037	$C_9H_8O_3$	0.190	0.0402	0.0559	0.0206	0.0330	0.0231	0.1010	0.0196	0.0115	p-Coumaric acid	Phenolic acid
163.061197	163.061175	$C_6H_{12}O_5$	0.136	0.0260	0.0067	0.0184	0.0241	0.0057	0.0112	0.0129	N.A	Rhamnose	Carbohydrate
165.040462	165.040436	$C_5H_{10}O_6$	0.156	1.1636	0.6665	10.3203	0.3194	0.0448	0.0806	0.0578	0.0709	Ribonic acid, Xylonic acid	Carbohydrate
165.055718	165.055686	$C_9H_{10}O_3$	0.191	0.0106	0.0082	0.0145	0.0221	0.0097	0.0323	0.0522	0.0184	3-Hydroxyphenylpropionic acid	Organic acid
165.092103	165.092096	$C_{10}H_{14}O_2$	0.046	N.A	N.A	0.0147	N.A	N.A	N.A	N.A	N.A	Perillic acid	Organic acid
167.034982	167.034984	$C_8H_8O_4$	-0.011	0.0276	0.0192	0.0589	0.0338	0.0177	0.0807	0.0575	0.0181	Vanillic acid	Phenolic acid
167.107753	167.107712	$C_{10}H_{16}O_2$	0.250	N.A	0.0000	0.0078	0.0137	N.A	N.A	N.A	N.A	Ascaridole	Terpenoid
169.014247	169.014232	$C_7H_6O_5$	0.086	0.0269	0.0145	N.A	0.0314	0.0660	0.0835	0.0311	0.0861	Gallic acid	Phenolic acid
171.029897	171.029886	$C_7H_8O_5$	0.066	0.0412	0.0552	0.0176	0.0442	0.0074	0.0252	0.0179	0.0419	Dehydroshikimic acid	Cyclitol
173.045547	173.045548	$C_7H_{10}O_5$	-0.002	3.6492	10.9577	0.1565	0.8624	2.5054	12.9735	0.2105	0.4625	Shikimic acid	Cyclitol
175.024812	175.024792	$C_6H_8O_6$	0.111	0.0593	0.0463	0.1291	0.0308	0.0113	0.0924	0.0183	0.0196	Ascorbic acid	Organic acid
177.019332	177.019358	$C_9H_6O_4$	-0.145	0.0039	0.0080	0.0073	N.A	0.0056	0.0217	0.0122	N.A	Aesculetin	Phenolic acid
179.056112	179.056103	$C_6H_{12}O_6$	0.049	3.8088	1.3235	8.8821	2.7235	0.3644	0.8338	1.3927	0.8881	Galactose, Glucose	Carbohydrate
179.071368	179.071362	$C_{10}H_{12}O_3$	0.032	0.0644	0.0183	0.0422	0.0701	0.0471	0.0786	0.1202	0.0951	Coniferyl alcohol	Phenolics

181.050632	181.050648	$C_9H_{10}O_4$	-0.089	0.0226	0.0227	0.0560	0.0298	0.1626	1.8826	0.7474	0.0400	3-Hydroxy-3-(3-hydroxyphenyl) propionic acid	Organic acid
181.071762	181.071767	$C_6H_{14}O_6$	-0.027	0.0293	0.0417	0.0895	0.0918	0.0257	0.0351	0.0806	0.0335	Mannitol	Carbohydrate
183.029897	183.029882	$C_8H_8O_5$	0.081	0.0216	0.0163	0.0416	0.0318	0.0294	0.0659	0.0320	N.A	4-O-Methylgallic acid	Phenolic acid
191.019726	191.019747	$C_6H_8O_7$	-0.109	0.1711	1.5996	0.2027	10.8449	0.0567	0.1475	0.0279	4.8480	Citric acid	Organic acid
191.056112	191.056111	$C_7H_{12}O_6$	0.005	22.9830	23.9673	11.6666	55.2903	14.7940	18.7235	1.5621	70.7379	Quinic acid	Organic acid/cyclitol
193.035376	193.035406	$C_6H_{10}O_7$	-0.153	0.1696	0.1088	0.7298	0.1675	0.0269	0.0660	0.0224	0.0427	Glucuronic acid	Carbohydrate
193.071762	193.071780	$C_7H_{14}O_6$	-0.095	0.4668	0.4796	3.4147	0.5316	0.3752	0.5123	0.8126	0.6198	Ononitol, pinitol	Cyclitol
195.051026	195.051049	$C_6H_{12}O_7$	-0.118	0.2000	0.1865	0.7258	0.2685	0.0131	0.0305	0.0130	0.0662	Gluconic acid	Carbohydrate
199.170354	199.170392	$C_{12}H_{24}O_2$	-0.193	0.4140	0.2453	0.8790	2.2403	0.4296	0.3511	0.6143	2.1537	Lauric acid	Fatty acid
201.113233	201.113250	$C_{10}H_{18}O_4$	-0.086	0.1163	2.0500	0.1011	0.2380	0.0835	0.1702	0.1652	0.3196	Sebacic acid	Organic acid
205.035376	205.035414	$C_7H_{10}O_7$	-0.186	0.2435	1.4875	1.0430	0.3199	0.1466	0.7364	0.1623	0.3518	Glucoascobic acid	Organic acid
205.087018	205.087077	$C_{12}H_{14}O_3$	-0.288	0.0152	0.0000	0.0243	N.A	N.A	N.A	N.A	N.A	Acetyl eugenol	Terpenoid
205.123403	205.123416	$C_{13}H_{18}O_2$	-0.061	N.A	0.0056	0.0024	N.A	0.0033	0.0090	N.A	0.0117	Phenyl ethyl-3-methyl butanote	Ester
207.051026	207.051051	$C_7H_{12}O_7$	-0.118	1.0587	0.5353	1.7670	0.5154	0.7563	0.5947	0.6399	0.4683	4-O-methyl-α-d-glucuronic acid	Carbohydrate
209.030291	209.030317	$C_6H_{10}O_8$	-0.123	N.A	0.0125	0.0059	3.1745	N.A	N.A	N.A	0.7817	D-glucaric acid	Carbohydrate
209.045547	209.045554	$C_{10}H_{10}O_5$	-0.032	0.0635	0.0429	N.A	0.0299	0.0591	0.5118	0.1427	0.0302	Hydroxyferulic acid	Phenolic acid
209.081932	209.081962	$C_{11}H_{14}O_4$	-0.142	N.A	0.0100	0.0166	0.0311	0.0138	0.0000	0.0355	0.0175	Sinapyl alcohol	Phenolics
209.154703	209.154741	$C_{13}H_{22}O_2$	-0.179	0.0093	0.0080	0.0168	0.0295	0.0131	0.0185	0.0127	0.0626	Isobornyl propionate, 3-methyl butanoic acid, 2- methyl butyrate	Terpenoid
225.076847	225.076847	$C_{11}H_{14}O_5$	0.000	0.0432	0.0992	0.0422	N.A	0.0454	0.1112	0.0405	0.0225	Dihydrosinapic acid	Phenolics
227.201654	227.201655	$C_{14}H_{28}O_2$	-0.006	0.8229	0.4212	0.4583	1.3523	0.8053	0.6180	1.1890	1.3747	Myristic acid	Fatty acid
245.081932	245.081964	$C_{14}H_{14}O_4$	-0.128	0.0061	N.A	0.0509	N.A	N.A	N.A	N.A	N.A	Dihydropiceatannol	Stilbenoid
253.092891	253.092894	$C_9H_{18}O_8$	-0.012	5.1403	1.5326	4.5794	2.5637	4.4624	2.8309	11.1923	1.1328	Galactosyl glycerol	Carbohydrate
255.066282	255.066473	$C_{15}H_{12}O_4$	-0.747	N.A	N.A	N.A	N.A	N.A	0.0091	N.A	N.A	Pinocembrin	Flavanone
269.045547	269.045701	$C_{15}H_{10}O_5$	-0.405	N.A	N.A	0.0082	N.A	N.A	N.A	N.A	N.A	Apigenin	Flavone
271.061197	271.061189	$C_{15}H_{12}O_5$	0.029	0.0139	0.0055	0.0168	N.A	0.0410	0.0445	0.0392	0.0331	Naringenin	Flavanone
271.082326	271.082336	$C_{12}H_{16}O_7$	-0.037	0.0997	0.0539	0.1260	0.0459	0.0896	0.1581	0.0841	0.0292	Arbutin	Glucoside
273.076847	273.076874	$C_{15}H_{14}O_5$	-0.099	0.0151	0.0066	0.0403	0.0172	0.0131	0.0338	0.0552	0.0141	Epiafzelechin	Flavan-3-ol
281.087806	281.087803	$C_{10}H_{18}O_9$	0.008	0.4188	0.1905	0.7979	0.2744	0.2242	0.1624	0.2973	0.0849	Xylobiose	Carbohydrate
285.040462	285.040449	$C_{15}H_{10}O_6$	0.046	0.0328	0.0409	0.0939	0.0678	0.2383	0.5172	0.1383	0.2533	Kaempferol	Flavonol
285.076847	285.076840	$C_{16}H_{14}O_5$	0.025	N.A	0.0074	0.0094	N.A	0.0432	0.0350	0.0122	N.A	Dihydrobiochanin A	Flavanone
285.113233	285.113211	$C_{17}H_{18}O_4$	0.078	N.A	N.A	0.0038	N.A	N.A	N.A	N.A	N.A	4'-Hydroxy-3,4,5-trimethoxystilbene	Stilbenoid
287.056112	287.056121	$C_{15}H_{12}O_6$	-0.031	0.0273	0.1005	0.1248	0.0208	0.0413	0.2302	0.0759	0.0172	Aromadendrin	Flavanonol
289.071762	289.071774	$C_{15}H_{14}O_6$	-0.042	0.6903	1.4823	9.9572	0.3815	0.8180	1.8374	4.4030	0.3523	Catechin	Flavan-3-ol
297.097976	297.097958	$C_{14}H_{18}O_7$	0.062	0.0586	0.4008	0.0557	0.0153	0.0522	0.4432	0.0292	0.0208	Picein	Phenolic glycoside

299.077241	299.077243	$C_{13}H_{16}O_8$	-0.006	0.3719	0.5648	0.2519	0.0689	0.3274	0.8861	0.1409	0.0721	4-Hydroxybenzoic acid 4-O-glucoside	Phenolic acid
299.165268	299.165248	$C_{19}H_{24}O_3$	0.0695	N.A	N.A	0.0026	0.0140	N.A	N.A	N.A	N.A	Z-bornyl-p-coumarate	Phenolics
299.201654	299.201618	$C_{20}H_{28}O_2$	0.119	0.3053	0.1607	0.1684	0.7876	1.5957	2.9240	0.9791	1.1685	Dehydroabietic acid	Terpenoid
301.035376	301.035357	$C_{15}H_{10}O_7$	0.064	0.0385	0.0221	0.2075	0.0378	0.2894	0.4068	0.2263	0.0882	Quercetin	Flavonol
301.092891	301.092851	$C_{13}H_{18}O_8$	0.135	0.1838	0.1563	0.1387	0.0660	0.0770	N.A	0.0608	0.0394	Tachioside	Phenolic glycoside
301.217304	301.217322	$C_{20}H_{30}O_2$	-0.061	0.1188	0.0747	0.4042	0.0883	19.5199	6.4120	38.3494	0.9515	Abietic acid	Terpenoid
303.051026	303.051039	$C_{15}H_{12}O_7$	-0.042	0.2077	0.2741	0.1098	0.2133	0.2900	0.3677	0.0854	0.1970	Taxifolin	Flavanonol
303.087412	303.087392	$C_{16}H_{16}O_6$	0.065	0.0180	0.0168	0.0176	N.A	0.0158	0.0358	0.0281	N.A	3'-O-methylcatechin	Flavan-3-ol
303.232954	303.232924	$C_{20}H_{32}O_2$	0.100	0.0809	0.0450	0.0586	0.2214	0.1584	0.0517	0.1614	0.3541	Anticopalic acid	Terpenoid
305.066676	305.066693	$C_{15}H_{14}O_7$	-0.055	0.8645	0.9429	1.3191	0.3084	0.9643	1.1187	0.3924	0.2984	Gallocatechol	Flavan-3-ol
305.248604	305.248586	$C_{20}H_{34}O_2$	0.059	0.0110	0.0051	0.0176	0.0249	0.1505	0.0540	0.0934	0.0458	18-Hydroxymanool	Terpenoid
311.222783	311.222757	$C_{18}H_{32}O_4$	0.085	0.0143	0.0072	0.0191	0.0210	0.0235	0.0229	0.0418	0.0215	Oxo-dihydroxyoctadecenoic acid	Fatty acid
315.072156	315.072167	$C_{13}H_{16}O_9$	-0.035	0.7287	1.6206	1.0104	0.1959	0.3659	1.8818	0.3741	0.1368	Protocatechuic acid 4-O-glucoside	Phenolic glycoside
315.108541	315.108536	$C_{14}H_{20}O_8$	0.016	0.1015	0.1186	0.0882	0.0311	0.0665	0.1790	0.0502	0.0267	3,4-Dimethoxyphenyl-beta-D-glucopyranoside	Phenolic glycoside
315.196568	315.196543	$C_{20}H_{28}O_3$	0.082	0.0193	0.0245	0.0960	0.1923	0.2243	0.3767	0.1724	0.0675	12-Hydroxydehydroabietic Acid	Terpenoid
317.030291	317.030288	$C_{15}H_{10}O_8$	0.011	0.0219	0.0361	0.0179	0.0369	0.2083	0.6980	0.0168	0.0802	Myricetin	Flavonoid
317.066676	317.066679	$C_{16}H_{14}O_7$	-0.007	0.0254	0.0117	0.1250	N.A	0.0319	0.0571	0.0498	N.A	Cedeodarin	Phenolics
317.212218	317.212192	$C_{20}H_{30}O_3$	0.082	0.0128	0.0139	0.0234	0.0362	0.3612	0.1082	0.2271	0.0318	3-oxoanticopalic Acid	Terpenoid
319.045941	319.045942	$C_{15}H_{12}O_8$	-0.004	0.0878	0.1798	0.0931	0.1243	0.1246	0.1891	0.0402	0.1068	Ampelopsin	Flavanonol
319.082326	319.082334	$C_{16}H_{16}O_7$	-0.024	0.0363	0.0325	0.0523	N.A	0.0486	0.3598	0.1146	N.A	4'-O-Methylepigallocatechin	Flavan-3-ol
319.227868	319.227853	$C_{20}H_{32}O_3$	0.047	0.0238	0.0067	1.1018	0.0205	0.7531	0.0939	21.1421	0.0311	Isocupressic/hydroxyanticopalic agatholic/imbricataloic acid	Terpenoid
321.082720	321.082739	$C_{12}H_{18}O_{10}$	-0.058	0.8884	1.1336	0.4021	0.0358	0.7773	1.8078	0.0433	0.0217	Lepidimoic acid	Carbohydrate
321.243519	321.243307	$C_{20}H_{34}O_3$	0.658	N.A	N.A	0.0118	N.A	N.A	N.A	N.A	N.A	Imbricatolic acid, Dihydroagatholic acid	Terpenoid
323.077241	323.077261	$C_{15}H_{16}O_8$	-0.062	0.0254	0.0161	0.0290	N.A	0.0198	0.0417	0.0302	0.0110	Skimmin	Phenolic glycoside
325.092891	325.092857	$C_{15}H_{18}O_8$	0.106	0.5181	0.4396	0.2439	0.0604	0.3178	0.5199	0.1166	0.0709	p-Coumaric acid glucoside	Phenolic glycoside
325.114020	325.114029	$C_{12}H_{22}O_{10}$	-0.027	1.3108	0.3616	0.5340	0.0720	0.4198	0.4374	0.2644	0.0567	Rutinose	Carbohydrate
327.123797	327.123871	$C_{19}H_{20}O_5$	-0.224	0.0047	N.A	0.0161	N.A	N.A	N.A	N.A	N.A	Demethoxylpinoresinol	Lignan
327.196568	327.196543	$C_{21}H_{28}O_3$	0.095	N.A	0.0044	N.A	N.A	N.A	N.A	0.0075	N.A	7-Oxodehydroabietic acid, methyl ester	Terpenoid
329.066676	329.066661	$C_{17}H_{14}O_7$	0.046	0.0104	0.0060	N.A	N.A	0.1170	0.0673	0.0526	N.A	Quercetin 3,4'-dimethyl ether	Flavonol
331.067070	331.067073	$C_{13}H_{16}O_{10}$	-0.008	0.2255	0.5936	0.1239	0.0373	0.1368	0.4031	0.0437	0.0377	Monogalloyl-glucose	Phenolic glycoside
331.155097	331.155066	$C_{19}H_{24}O_5$	0.096	0.0063	0.0057	0.0050	N.A	0.0079	0.0129	N.A	N.A	Gibberellin A20	Terpenoid
331.176227	331.176240	$C_{16}H_{28}O_7$	-0.041	0.3207	0.1535	0.3128	0.0666	0.2229	0.1796	0.0667	0.0583	2-Hydroxy-1,8-cineole-glucopyranoside	Terpene glycoside
333.207133	333.207101	$C_{20}H_{30}O_4$	0.095	0.0469	0.0752	0.1099	0.0532	1.2129	0.8122	0.1660	0.4949	Dehydropinifolic acid	Terpenoid
335.222783	335.222752	$C_{20}H_{32}O_4$	0.093	0.0976	0.0173	0.2265	0.0422	3.5120	0.0538	0.1899	0.0707	Pinifolic acid	Terpenoid

337.092891	337.092906	$C_{16}H_{18}O_8$	-0.045	0.3317	5.2135	0.9184	0.2923	0.1841	9.0672	0.4875	0.1254	3-p-Coumaroylquinic acid	Phenolic acid
339.072156	339.072152	$C_{15}H_{16}O_9$	0.010	0.0251	0.0150	N.A	N.A	0.0248	0.0431	0.0111	N.A	Aesculin	Phenolic glycoside
341.087806	341.087840	$C_{15}H_{18}O_9$	-0.102	0.1227	0.4250	0.6781	N.A	0.1105	0.4676	0.0772	0.0240	Glucocaffeic acid	Phenolic glycoside
341.108935	341.108930	$C_{12}H_{22}O_{11}$	0.014	1.5522	0.9974	N.A	1.2741	1.7211	0.9897	2.0063	0.5898	Sucrose	Carbohydrate
341.124191	341.124179	$C_{16}H_{22}O_8$	0.035	0.0604	0.0395	N.A	N.A	0.0562	0.0840	0.0715	0.0143	Coniferin	Phenolic glycoside
343.082326	343.082251	$C_{18}H_{16}O_7$	0.220	0.0175	N.A	0.0193	N.A	N.A	N.A	N.A	N.A	Cirsilineol	Flavone
343.139841	343.139842	$C_{16}H_{24}O_8$	-0.001	0.1100	0.0925	N.A	0.0189	0.0738	0.1377	0.0658	0.0233	Dihydroconiferin	Phenolic glycoside
345.061591	345.061568	$C_{17}H_{14}O_8$	0.068	0.0296	0.0097	0.0751	0.0124	0.0750	0.0375	0.0247	0.0106	Syringetin	Flavonol
345.134362	345.134375	$C_{19}H_{22}O_6$	-0.037	0.2161	0.0363	0.1291	N.A	0.0319	0.0350	0.0439	N.A	Taxiresinol	Lignan
345.207133	345.207130	$C_{21}H_{30}O_4$	0.010	0.0070	N.A	N.A	0.0317	N.A	N.A	N.A	N.A	Pinusolide	Terpenoid
349.238433	349.238470	$C_{21}H_{34}O_4$	-0.107	0.1325	0.0000	0.0084	0.0193	20.2415	0.0136	0.2060	0.0233	Pinifolate, Nor-16-acetylimbricatolic	Terpenoid
353.087806	353.087826	$C_{16}H_{18}O_9$	-0.057	0.0389	0.1067	0.2813	N.A	0.0290	0.1271	0.0292	N.A	Chlorogenic acid	Phenolic acid
353.108935	353.108962	$C_{13}H_{22}O_{11}$	-0.076	18.1855	21.4535	0.9814	0.6479	3.5868	9.1686	0.1742	0.3340	Methylene sucrose	Carbohydrate
355.067070	355.067101	$C_{15}H_{16}O_{10}$	-0.085	0.0112	0.0088	0.0091	0.0117	0.0067	0.0142	N.A	N.A	Caffeic acid 3-O-glucuronide	Phenolic glycoside
355.103456	355.103455	$C_{16}H_{20}O_9$	0.001	0.4056	0.4255	3.0177	0.0380	0.3406	0.5210	0.3050	0.0280	1-O-Feruloyl-beta-D-glucose	Phenolic glycoside
357.082720	357.082686	$C_{15}H_{18}O_{10}$	0.096	0.0377	0.0343	0.0516	N.A	0.0364	0.0489	0.0107	N.A	Dihydrocaffeic acid 3-O-glucuronide	Phenolic glycoside
357.119106	357.119101	$C_{16}H_{22}O_9$	0.014	0.1576	1.6374	0.2719	0.0454	0.1400	2.0615	0.0548	0.0177	Junipediol B 8-glucoside	Phenolic glycoside
357.134362	357.134403	$C_{20}H_{22}O_6$	-0.115	0.0130	N.A	0.0230	N.A	N.A	N.A	N.A	N.A	Pinoresinol	Lignan
359.077241	359.077239	$C_{18}H_{16}O_8$	0.005	0.0164	0.0120	0.0468	N.A	0.0235	0.0285	0.0152	N.A	Irigenin	Flavone
359.134756	359.134738	$C_{16}H_{24}O_9$	0.050	0.1604	0.0788	0.5916	0.0295	0.0854	0.1072	0.1592	0.0195	Junipediol A 8-glucoside	Phenyl propanoid
359.150012	359.150016	$C_{20}H_{24}O_6$	-0.012	N.A	0.0081	N.A	0.0115	0.0078	0.0127	N.A	N.A	Lariciresinol	Lignan
359.222783	359.222765	$C_{22}H_{32}O_4$	0.030	0.0252	0.0061	0.0097	0.0212	0.1211	0.1185	0.0907	0.0102	7-Acetoxyabietic acid	Terpenoid
361.165662	361.165663	$C_{20}H_{26}O_6$	-0.003	0.0205	0.0159	0.0296	0.0250	0.0142	0.0308	0.0169	N.A	Secoisolariciresinol	Lignan
361.238433	361.238418	$C_{22}H_{34}O_4$	0.042	0.0132	0.0000	0.0083	N.A	N.A	N.A	N.A	N.A	Acetoxyanticopalic, Acetylisocupressic acid	Terpenoid
363.254083	363.254092	$C_{22}H_{36}O_4$	-0.025	0.0110	0.0039	N.A	0.0136	0.4605	0.0513	0.3891	N.A	Acetylimbricatolic acid	Terpenoid
367.103456	367.103455	$C_{17}H_{20}O_9$	0.002	0.1194	0.2885	0.0976	0.0267	0.0774	0.4243	0.0247	0.0106	3-Feruloylquinic acid	Phenolic acid
369.082720	369.082669	$C_{16}H_{18}O_{10}$	0.141	0.0265	N.A	0.0676	N.A	N.A	N.A	N.A	N.A	Ferulic acid 4-O-glucuronide	Phenolic glycoside
371.098370	371.098371	$C_{16}H_{20}O_{10}$	0.000	0.0511	0.0610	0.0702	N.A	0.0408	0.0822	0.0106	N.A	Dihydroferulic acid 4-O-glucuronide	Phenolic glycoside
371.119500	371.119486	$C_{13}H_{24}O_{12}$	0.037	19.1510	8.2637	9.8507	8.7571	6.9002	3.1312	0.4502	4.2103	Galactosyl pinitol	Cyclitol
371.134756	371.134716	$C_{17}H_{24}O_9$	0.109	0.1323	0.1029	0.0752	0.0264	N.A	N.A	N.A	N.A	Syringin	Phenolic glycoside
373.092891	373.092880	$C_{19}H_{18}O_8$	0.029	0.0158	0.0099	0.0430	N.A	0.0163	N.A	0.0137	N.A	Junipegenin C	Flavone
375.144927	375.144909	$C_{20}H_{24}O_7$	0.046	0.0134	N.A	0.0210	N.A	N.A	N.A	N.A	N.A	Todolactol A	Lignan
385.092891	385.092937	$C_{20}H_{18}O_8$	-0.120	0.0289	0.0076	0.4560	N.A	0.0103	0.0183	N.A	N.A	Diferulic acid	Phenolic acid
385.150406	385.150404	$C_{18}H_{26}O_9$	0.006	0.2092	0.0589	0.0418	0.0412	0.1420	0.1096	0.0240	0.0227	Juniperoside	Phenolic glycoside

385.186791	385.186843	$C_{19}H_{30}O_{8}$	-0.134	0.0625	0.0466	0.1712	0.0146	0.0526	0.0805	0.0312	0.0122	Junipeionoloside, Corchoionoside C	Terpene glycoside
387.144927		$C_{21}H_{24}O_{7}$		N.A	N.A	0.0077	N.A	0.0057	0.0120	0.0061	N.A	Dihydrosamidin	Benzopyranone
389.124191	389.124199	$C_{20}H_{22}O_{8}$	-0.020	0.0433	0.0520	N.A	N.A	0.0438	0.0809	0.0197	N.A	Piceid	Stilbenoid
389.145321	389.145332	$C_{17}H_{26}O_{10}$	-0.030	0.0869	0.0495	0.2072	N.A	0.1008	0.1107	0.0938	N.A	Loganin	Terpenoid
389.160577	389.160579	$C_{21}H_{26}O_{7}$	-0.005	0.0061	N.A	0.0301	N.A	N.A	N.A	N.A	N.A	5'-Methoxylariciresinol	Lignan
397.114020	397.113982	$C_{18}H_{22}O_{10}$	0.099	0.0205	0.0131	0.0439	N.A	0.0208	0.0217	0.0097	N.A	3-Sinapoylquinic acid	Phenolic acid
397.129277	397.129217	$C_{22}H_{22}O_{7}$	0.150	N.A	N.A	0.0046	N.A	N.A	N.A	N.A	N.A	deoxypodophyllotoxin	Lignan
399.217698	399.217642	$C_{24}H_{32}O_{5}$	0.140	0.0179	0.0059	0.0408	N.A	0.0284	0.0644	0.0109	N.A	Succinyl 7-oxodehydroabietol	Terpenoid
405.119106	405.119090	$C_{20}H_{22}O_{9}$	0.039	0.0208	0.7188	0.0670	0.0370	0.0257	0.9813	0.0224	N.A	Astringin	Stilbenoid
415.103456	415.103347	$C_{21}H_{20}O_{9}$	0.262	0.0164	0.0000	0.1250	N.A	N.A	N.A	N.A	N.A	Apigenin 7-rhamnoside	Flavonoid
417.082720	417.082706	$C_{20}H_{18}O_{10}$	0.034	0.0528	0.0186	0.1706	N.A	0.0118	0.0429	0.0445	N.A	Salvianolic acid G	Coumaric acid
417.155491	417.155517	$C_{22}H_{26}O_{8}$	-0.060	0.0047	N.A	0.0174	N.A	N.A	N.A	N.A	N.A	Syringaresinol	Lignan
419.243912	419.243864	$C_{24}H_{36}O_{6}$	0.115	0.0045	0.0180	0.0040	N.A	N.A	N.A	N.A	N.A	Succinylisocupresic, succinylagatholic	Terpenoid
429.373804	429.373750	$C_{29}H_{50}O_{2}$	0.126	N.A	N.A	N.A	N.A	0.0193	0.0155	0.0269	N.A	β-Tocopherol	Lipid
431.098370	431.098351	$C_{21}H_{20}O_{10}$	0.046	0.0516	0.0369	0.2117	0.0350	0.0407	0.0583	0.0460	0.0303	Kaempferol-3-O-rhamnoside	Flavonoid
433.077635	433.077610	$C_{20}H_{18}O_{11}$	0.058	0.0253	0.0161	0.1527	N.A	0.0172	0.0125	0.0559	N.A	Quercetin 3-O-arabinoside	Flavonoid
433.114020	433.114105	$C_{21}H_{22}O_{10}$	-0.195	0.0748	0.0511	0.1844	0.0210	0.0442	0.0547	0.0327	0.0166	Aromadendrin 7-O-rhamnoside, Naringenin glucoside	Flavonoid
435.129671	435.129692	$C_{21}H_{24}O_{10}$	-0.050	0.0311	0.0463	0.0877	N.A	0.0298	0.0972	0.0324	N.A	Phloridzin	Phenolic glycoside
435.296342	435.296324	$C_{22}H_{44}O_{8}$	0.040	2.1306	0.5119	1.0916	0.2507	0.3753	0.1812	0.2703	0.1789	Sorbitol palmitate	Polysorbate
447.093285	447.093306	$C_{21}H_{20}O_{11}$	-0.047	0.1627	0.1063	0.2995	0.2887	0.1462	0.1508	0.0376	0.9551	Astragalin, Quercitrin, Kaempferol 7-O-glucoside	Flavonoid
449.072550	449.072638	$C_{20}H_{18}O_{12}$	-0.197	0.0072	N.A	N.A	N.A	N.A	N.A	N.A	N.A	Myricetin 3-O-arabinoside	Flavonoid
449.108935	449.108971	$C_{21}H_{22}O_{11}$	-0.079	0.0540	0.0321	0.3649	N.A	0.0359	0.0517	0.0596	N.A	Taxifolin 7-O-rhamnoside	Flavonoid
451.124585	451.124593	$C_{21}H_{24}O_{11}$	-0.018	0.0567	0.0666	0.3779	0.0127	0.0496	0.2356	0.1262	N.A	Catechin-3-glucoside	Flavonoid glucoside
457.077635	457.077640	$C_{22}H_{18}O_{11}$	-0.012	0.0083	0.0052	0.0172	N.A	0.0078	0.0171	N.A	N.A	Gallocatechin 3-O-gallate	Flavonoid
459.093285	459.093349	$C_{22}H_{20}O_{11}$	-0.141	0.0171	0.0061	0.1048	N.A	0.0162	0.0534	0.0059	N.A	Apigenin 7-O-glucuronide methyl ester	Flavonoid
461.108935	461.108912	$C_{22}H_{22}O_{11}$	0.051	0.0907	0.0304	N.A	N.A	0.0829	0.0556	0.0174	N.A	Chrysoeriol 7-O-glucoside, Isorhamnetin 3-rhamnoside	Flavonoid
463.088200	463.088211	$C_{21}H_{20}O_{12}$	-0.024	0.1793	0.0364	0.4548	0.0410	0.1968	0.0607	0.0737	0.0988	Isoquercetin	Flavonoid
463.327642	463.327615	$C_{24}H_{48}O_{8}$	0.058	2.0250	0.4219	1.4100	0.2487	0.6189	0.2589	0.3058	0.2092	Mannitol stearate	Fatty acid ester
463.342898	463.342757	$C_{28}H_{48}O_{5}$	0.305	0.0077	0.0000	N.A	N.A	N.A	N.A	N.A	N.A	Castasterone	Flavonoid glucoside
465.103850	465.103841	$C_{21}H_{22}O_{12}$	0.019	0.0893	0.0576	0.2496	N.A	0.1147	0.0653	0.0314	0.0179	Taxifolin 7-O-beta-D-glucopyranoside	Flavonoid
471.093285	471.093401	$C_{23}H_{20}O_{11}$	-0.161	0.0169	N.A	0.0420	0.0149	N.A	N.A	N.A	N.A	4''-O-Methylepigallocatechin 3-O-gallate	Flavonoid
473.108935	473.108940	$C_{23}H_{22}O_{11}$	-0.011	0.0088	N.A	0.0395	N.A	N.A	N.A	N.A	N.A	6''-O-Acetylgenistin, Apigenin 7-(6''-acetylglucoside)	Flavonoid
477.067464	477.067246	$C_{21}H_{18}O_{13}$	0.458	N.A	0.0063	0.0054	N.A	N.A	N.A	N.A	N.A	Quercetin 3'-O-glucuronide	Flavonoid

477.103850	477.103814	$C_{22}H_{22}O_{12}$	0.075	0.1410	0.1304	0.2986	0.0197	0.1330	0.1535	0.0181	0.0401	Isorhamnetin 3-glucoside, 3′-O-methylquercetin 3-glucoside	Flavonoid glucoside
477.176621	477.176619	$C_{24}H_{30}O_{10}$	0.004	0.0230	0.0106	0.0541	N.A	0.0196	0.0215	0.0125	N.A	Junipercomnoside A	Flavonoid glucoside
479.083114	479.083093	$C_{21}H_{20}O_{13}$	0.045	0.0429	N.A	0.0241	N.A	0.0684	0.0170	0.0097	0.0290	Myricetin 3-O-glucoside	Flavonoid
479.337813	479.337831	$C_{28}H_{48}O_{6}$	-0.037	0.0053	0.0055	0.0058	N.A	0.0190	0.0094	0.0105	N.A	Brassinolide	Flavonoid glucoside
481.098764	481.098705	$C_{21}H_{22}O_{13}$	0.124	0.0255	0.0154	N.A	N.A	0.0338	0.0206	0.0061	N.A	Ampelopsin 7-glucoside	Flavonoid glucoside
489.103850	489.103832	$C_{23}H_{22}O_{12}$	0.037	0.0599	0.0046	0.0316	0.0178	0.0696	0.0183	N.A	0.0399	Kaempferol 3-(6''-acetylglucoside)	Flavonoid glucoside
493.098764	493.098749	$C_{22}H_{22}O_{13}$	0.031	0.0563	0.0720	0.0479	N.A	0.0736	0.1062	0.0103	0.0200	Laricitrin 3-glucoside	Flavonoid glucoside
503.161758	503.161774	$C_{18}H_{32}O_{16}$	-0.031	0.0865	0.1998	0.6013	0.0461	0.1517	0.1266	0.1511	0.0289	Raffinose	Carbohydrate
505.098764	505.098822	$C_{23}H_{22}O_{13}$	-0.115	0.2963	N.A	0.0783	N.A	0.4589	N.A	0.0253	N.A	Quercetin 3-(6''-acetylglucoside)	Flavonoid glucoside
505.135150	505.135130	$C_{24}H_{26}O_{12}$	0.040	0.0123	0.0151	0.0419	N.A	0.0186	0.0384	0.0097	N.A	Junipegenin B 7-O-glucoside	Flavonoid glucoside
505.207921	505.207826	$C_{26}H_{34}O_{10}$	0.189	N.A	0.0060	0.0930	N.A	N.A	0.0120	0.0179	N.A	Icariside E4	Flavonoid glucoside
507.114414	507.114417	$C_{23}H_{24}O_{13}$	-0.004	N.A	0.0085	0.0331	N.A	N.A	0.0279	0.0073	N.A	Syringetin 3-glucoside	Flavonoid glucoside
507.187185	507.187207	$C_{25}H_{32}O_{11}$	-0.043	0.0685	0.0422	0.2487	N.A	0.0475	0.0441	0.0174	0.0152	Gibberellin 2-O-beta-D-glucoside	Flavonoid glucoside
519.114414	519.114472	$C_{24}H_{24}O_{13}$	-0.111	N.A	0.0070	0.0229	N.A	0.0880	0.0142	N.A	0.0158	Isorhamnetin 3-(6''-acetylglucoside)	Flavonoid glucoside
521.093679	521.093589	$C_{23}H_{22}O_{14}$	0.171	0.0144	N.A	0.0034	N.A	0.0212	N.A	N.A	N.A	Myricetin 3-(6″-acetyl-glucoside)	Flavonoid glucoside
521.130064	521.130076	$C_{24}H_{26}O_{13}$	-0.022	0.0103	0.0106	0.0225	N.A	0.0149	0.0277	N.A	N.A	Iridin	Flavonoid glucoside
521.202835	521.202915	$C_{26}H_{34}O_{11}$	-0.153	0.0830	0.0351	0.1077	N.A	0.0671	0.0451	0.0237	N.A	Lariciresinol glycoside	Lignan
533.202835	533.202855	$C_{27}H_{34}O_{11}$	-0.036	0.0074	0.0081	N.A	N.A	N.A	N.A	N.A	N.A	Arctin	Lignan
535.109329	535.109337	$C_{24}H_{24}O_{14}$	-0.016	0.0147	N.A	0.0088	N.A	0.0155	N.A	N.A	N.A	Laricitrin 3-(6''-acetylglucoside)	Flavonoid glucoside
537.082720	537.082672	$C_{30}H_{18}O_{10}$	0.091	0.0043	0.0000	0.0825	N.A	0.0047	N.A	1.5119	N.A	sennidin A, Cupressuflavone	Flavonoid
549.124979	549.125086	$C_{25}H_{26}O_{14}$	-0.195	0.0114	N.A	0.0382	N.A	0.0136	N.A	N.A	N.A	Syringetin 3-(6''-acetylglucoside)	Flavonoid glucoside
551.098370	551.098297	$C_{31}H_{20}O_{10}$	0.134	N.A	N.A	0.0069	N.A	N.A	N.A	0.3289	N.A	Bilobetin	Flavonoid glucoside
555.223571	555.223586	$C_{30}H_{36}O_{10}$	-0.028	0.0064	0.0087	0.0273	N.A	0.0094	N.A	0.0170	N.A	Lariciresinol-sesquilignan	Lignan
563.140629	563.140640	$C_{26}H_{28}O_{14}$	-0.020	0.0061	N.A	0.0368	N.A	N.A	N.A	N.A	N.A	Apigenin 7-O-apiosyl-glucoside	Flavonoid glucoside
575.119500	575.119578	$C_{30}H_{24}O_{12}$	-0.135	N.A	0.0045	N.A	N.A	0.0046	0.0130	N.A	N.A	Procyanidin A2	Tannins
577.135150	577.135160	$C_{30}H_{26}O_{12}$	-0.017	0.0240	0.2653	0.8991	N.A	0.0274	0.1327	0.2030	0.0292	Procyanidin B1	Tannins
577.156279	577.156229	$C_{27}H_{30}O_{14}$	0.087	0.0065	0.0099	0.0144	0.3474	0.0042	0.0153	N.A	0.3937	Isorhoifolin, Isovitexin rhamnoside	Flavonoid glucoside
579.171929	579.172001	$C_{27}H_{32}O_{14}$	-0.124	0.0086	0.6968	0.0316	0.0255	0.0079	1.2774	N.A	0.0171	Naringin	Flavonoid glucoside
593.130064	593.130163	$C_{30}H_{26}O_{13}$	-0.166	0.1088	0.1425	0.3786	0.0306	0.1098	0.1187	0.0326	0.0281	Kaempferol 3-(6″-coumaroyl-glucoside), Epigallocatechin-(4)-catechin	Flavonoid
593.151194	593.151248	$C_{27}H_{30}O_{15}$	-0.091	0.0160	0.0242	0.2781	N.A	0.0046	0.0280	0.0760	0.0177	Kaempferol 3-O-rutinoside	Flavonoid
595.130458	595.130708	$C_{26}H_{28}O_{16}$	-0.420	0.0106	N.A	0.0189	N.A	N.A	N.A	N.A	N.A	Quercetin 3-vicianoside	Flavonoid
595.166844	595.166960	$C_{27}H_{32}O_{15}$	-0.195	0.0086	0.0138	0.0525	N.A	0.0073	0.0204	N.A	N.A	Eriocitrin	Flavonoid glucoside
607.109329	607.109419	$C_{30}H_{24}O_{14}$	-0.147	0.0115	0.0074	0.0188	N.A	0.0055	N.A	N.A	N.A	Prodelphinidin A	Tannins
609.124979	609.125018	$C_{30}H_{26}O_{14}$	-0.064	0.0838	0.0143	0.0646	N.A	0.0820	0.0128	N.A	N.A	Prodelphinidin B	Tannins

609.146108	609.146205	$C_{27}H_{30}O_{16}$	-0.159	0.0158	0.0141	4.8144	0.0144	0.0060	0.0219	0.5608	N.A	Rutin	Flavonoid
623.161758	623.161783	$C_{28}H_{32}O_{16}$	-0.040	0.0124	0.0202	N.A	N.A	0.0084	0.0280	0.0173	N.A	Isorhamnetin-3-O-rutinoside	Flavonoid
623.198144	623.198087	$C_{29}H_{36}O_{15}$	0.092	0.0037	0.0183	N.A	N.A	0.0055	0.0316	N.A	N.A	Verbascoside	Phenolic glycoside
625.141023	625.141258	$C_{27}H_{30}O_{17}$	-0.376	0.0094	0.0070	0.0415	N.A	0.0071	0.0153	N.A	N.A	Myricetin 3-O-rutinoside	Flavonoid
639.135544	639.135719	$C_{31}H_{28}O_{15}$	-0.274	0.0216	N.A	N.A	N.A	N.A	N.A	N.A	N.A	Quercetin 3-(6''-ferulylglucoside)	Flavonoid
639.156673	639.156876	$C_{28}H_{32}O_{17}$	-0.318	0.0155	0.0220	N.A	N.A	0.0181	0.0251	N.A	0.0788	Laricitrin 3-rutinoside	Flavonoid
653.172323	653.172305	$C_{29}H_{34}O_{17}$	0.028	0.0080	N.A	0.0176	N.A	0.0072	N.A	N.A	N.A	Syringetin 3-rutinoside	Flavonoid
665.214700	665.214582	$C_{24}H_{42}O_{21}$	-0.177	0.0218	0.0252	0.0279	N.A	0.0019	0.0120	0.0027	N.A	Stachyose	Carbohydrate
667.260744	667.260675	$C_{32}H_{44}O_{15}$	0.104	0.0111	0.0184	0.0168	N.A	0.0083	N.A	N.A	N.A	Junipercomnoside B	Flavonoid glucoside
739.166844	739.166859	$C_{39}H_{32}O_{15}$	-0.020	N.A	N.A	N.A	N.A	0.0400	0.0444	N.A	N.A	Kaempferol 3-(3,6-di-coumaroyl-glucoside)	Flavonoid glucoside
755.204017	755.203999	$C_{33}H_{40}O_{20}$	0.024	N.A	N.A	0.0065	N.A	N.A	N.A	N.A	N.A	Quercetin 3-rutinoside-7-rhamnoside	Flavonoid
771.198932	771.198937	$C_{33}H_{40}O_{21}$	-0.006	N.A	N.A	0.0066	N.A	N.A	N.A	N.A	N.A	Kaempferol 3,7,4'-O-triglucoside	Flavonoid
865.198538	865.199021	$C_{45}H_{38}O_{18}$	-0.559	N.A	0.0554	0.0594	N.A	N.A	N.A	0.0133	N.A	Procyanidin trimer C1	Tannins

Table S2. Compounds identified with (+) APPI from the conifer extracts

Calculated *m/z*	Observed *m/z*	Elemental formula	Error (ppm)	Ion(s)*	Relative Intensity (%)								Tentative compound(s)	Class
					Water				Ethanol					
					Pine	*Spruce*	*Juniper*	*Larch*	*Pine*	*Spruce*	*Juniper*	*Larch*		
111.044056	111.044032	$C_6 H_6 O_2$	0.215	$[M + H]^{+*}$, $M^{+\bullet}$	0.0195	0.0239	0.0088	0.0972	N.A	0.0119	0.0106	0.0436	Catechol	Phenol
123.044056	123.044049	$C_7 H_6 O_2$	0.053	$[M + H]^{+*}$, $M^{+\bullet}$	0.0548	0.1362	0.0404	0.0593	0.0116	0.0450	0.0659	0.0416	Benzoic acid	Phenolic acid
125.059706	125.059699	$C_7 H_8 O_2$	0.058	$[M + H]^{+*}$, $M^{+\bullet}$	0.0450	0.0451	0.0146	0.0509	0.0173	0.0191	0.0645	0.0451	4-Methylcatechol	Phenolics
127.038971	127.038964	$C_6 H_6 O_3$	0.049	$[M + H]^{+*}$, $M^{+\bullet}$	0.1371	0.0821	0.0912	0.2023	0.0205	0.0460	0.0412	0.0738	Pyrogallol	Phenol
135.080441	135.080427	$C_9 H_{10} O$	0.109	$[M + H]^{+*}$, $M^{+\bullet}$	0.0133	0.0587	0.0080	0.0497	0.0057	0.0157	0.0163	0.0189	Cinnamyl alcohol	Phenol
135.116827	135.116816	$C_{10} H_{14}$	0.084	$[M + H]^{+*}$, $M^{+\bullet}$	0.0373	0.0160	0.0132	0.0137	0.0366	0.0379	0.0291	0.0228	Verbenene	Terpenoid
137.059706	137.059699	$C_8 H_8 O_2$	0.054	$[M + H]^{+*}$, $M^{+\bullet}$	0.1055	0.7745	0.0484	0.0435	0.0297	0.7503	0.0615	0.0288	Piceol	Phenolics
137.132477	137.132465	$C_{10} H_{16}$	0.084	$[M + H]^{+*}$, $M^{+\bullet}$	0.0177	0.0099	0.0099	0.0202	0.0359	0.0824	0.0344	0.0192	Monoterpene	Terpenoid
138.127726	138.127699	$C_9 H_{15} N$	0.192	[M + H]+	N.A	0.0042	N.A	N.A	N.A	N.A	N.A	N.A	1,6 hydropinidine	alkaloids
139.038971	139.038970	$C_7 H_6 O_3$	0.005	$[M + H]^{+*}$, $M^{+\bullet}$	0.1859	0.2130	0.0819	0.4905	0.0289	0.2739	0.0505	0.1594	Salicyclic acid	Phenolic acid
139.075356	139.075340	$C_8 H_{10} O_2$	0.117	$[M + H]^{+*}$, $M^{+\bullet}$	1.0657	0.2786	N.A	0.3157	N.A	N.A	N.A	N.A	Tyrosol	Phenolics
139.135551	139.135536	$C_9 H_{16} N$	0.108	$[M + H]^{+}$	N.A	0.0043	N.A	N.A	N.A	N.A	N.A	N.A	Pinidine	Alkaloids
147.044056	147.044047	$C_9 H_6 O_2$	0.059	$[M + H]^{+*}$, $M^{+\bullet}$	0.0648	0.0667	0.0255	0.0537	0.0230	0.2722	0.0269	0.0379	Coumarin	Phenolic acid
149.059706	149.059717	$C_9 H_8 O_2$	-0.073	$[M + H]^{+*}$, $M^{+\bullet}$	0.0427	0.0569	0.0222	N.A	0.0136	0.0744	0.0267	0.0215	Cinnamic acid	Organic acid
151.075356	151.075381	$C_9 H_{10} O_2$	-0.163	$[M + H]^{+*}$, $M^{+\bullet}$	N.A	N.A	0.0251	N.A	N.A	N.A	N.A	N.A	4-hydroxycinnamyl alcohol	Phenolics
151.111742	151.111744	$C_{10} H_{14} O$	-0.017	$[M + H]^{+*}$, $M^{+\bullet}$	0.0369	0.0205	0.0140	0.0150	N.A	0.0413	0.0241	0.0192	Thymol	Terpenoid
153.054621	153.054630	$C_8 H_8 O_3$	-0.062	$[M + H]^{+*}$, $M^{+\bullet}$	0.2729	0.2641	0.0574	0.2139	0.0415	0.6544	0.0508	0.0728	Vanillin	Organic acid
153.127392	153.127404	$C_{10} H_{16} O$	-0.080	$[M + H]^{+*}$, $M^{+\bullet}$	0.0854	0.0250	0.0145	0.0176	0.0215	0.0646	0.0324	0.0181	Methyl thymol, Thujone, Piperitone	Terpenoid
154.122641	154.122638	$C_9 H_{15} N O$	0.018	$[M + H]^{+}$	N.A	0.0159	N.A	N.A	N.A	N.A	N.A	N.A	1,6-dehydropinidinone	Alkaloid
155.033885	155.033901	$C_7 H_6 O_4$	-0.101	$[M + H]^{+*}$, $M^{+\bullet}$	0.1168	0.0271	0.0267	0.1072	0.0115	0.0483	0.0180	0.0353	Protocatechuic acid	Phenolic acid
155.070271	155.070266	$C_8 H_{10} O_3$	0.029	$[M + H]^{+*}$, $M^{+\bullet}$	0.0416	0.0088	0.0143	0.0211	0.0175	0.0540	0.0375	0.0237	Sylvopinol, Hydroxytyrosol	Phenolics
156.138291	156.138281	$C_9 H_{17} N O$	0.062	$[M + H]^{+}$	N.A	0.0259	N.A	N.A	N.A	N.A	N.A	N.A	epipinidinone	alkaloids
159.137956	159.137965	$C_9 H_{18} O_2$	-0.051	$[M + H]^{+}$	N.A	N.A	N.A	N.A	N.A	0.0031	0.0117	N.A	Nonanoic acid	Fatty acid
163.038971	163.038963	$C_9 H_6 O_3$	0.047	$[M + H]^{+*}$, $M^{+\bullet}$	0.0170	0.1002	0.0579	0.0440	0.0106	0.1523	N.A	0.0233	Hydroxycinnamate	Phenolic acid
163.075356	163.075352	$C_{10} H_{10} O_2$	0.022	$[M + H]^{+*}$, $M^{+\bullet}$	0.1266	0.0543	0.1583	0.0275	0.1218	0.3656	0.1254	0.0215	Safrole, Methyl cinnamate	Phenolic acid
164.046796	164.046810	$C_9 H_8 O_3$	-0.087	$[M + H]^{+}$, $M^{+\bullet *}$	0.1461	0.1906	0.0384	0.1027	0.0161	0.3634	0.0285	0.0511	p-coumaric acid	Phenolic acid

165.091006	165.091009	$C_{10}H_{12}O_2$	-0.018	$[M + H]^{+*}$, $M^{+\bullet}$	0.0494	0.0200	0.0224	0.0221	0.0320	0.1220	0.0389	0.0152	2-phenyl ethyl acetate, Eugenol	Terpenoid
165.127392	165.127399	$C_{11}H_{16}O$	-0.044	$[M + H]^{+*}$, $M^{+\bullet}$	0.0102	0.0062	0.0047	0.0112	0.0109	0.0224	N.A	0.0163	Thymol methyl ether	Terpenoid
167.070271	167.070285	$C_9H_{10}O_3$	-0.087	$[M + H]^{+}$, $M^{+\bullet}$	0.0323	0.0180	0.0243	0.0158	0.0130	0.0907	0.0287	0.0234	3-Hydroxyphenylpropionic acid	Phenolic acid
167.106656	167.106663	$C_{10}H_{14}O_2$	-0.044	$[M + H]^{+*}$, $M^{+\bullet}$	0.0391	0.0189	0.0210	0.0241	0.0227	0.0662	0.0395	0.0177	Perillic acid	Organic acid
169.049535	169.049540	$C_8H_8O_4$	-0.028	$[M + H]^{+*}$, $M^{+\bullet}$	0.0630	0.0111	0.0270	0.0270	0.0201	0.0869	0.0258	0.0104	Vanillic acid	Phenolic acid
169.122306	169.122322	$C_{10}H_{16}O_2$	-0.094	$[M + H]^{+*}$, $M^{+\bullet}$	0.0466	0.0151	0.0118	0.0172	0.0164	0.0397	0.0305	0.0143	Ascaridole	Terpenoid
171.028800	171.028808	$C_7H_6O_5$	-0.049	$[M + H]^{+*}$, $M^{+\bullet}$	0.0190	0.0046	0.0042	0.0172	0.0045	0.0095		0.0130	Gallic acid	Phenolic acid
173.044450	173.044454	$C_7H_8O_5$	-0.026	$[M + H]^{+*}$, $M^{+\bullet}$	0.0288	0.0102	0.0127	0.0301	0.0052	0.0138	0.0056	0.0075	Dehydroshikimic acid	Terpenoid
175.060100	175.060118	$C_7H_{10}O_5$	-0.099	$[M + H]^{+*}$, $M^{+\bullet}$	0.0148	0.0036	N.A	N.A	N.A	N.A	N.A	N.A	Shikimic acid	Cyclitol
176.070605	176.070600	$C_{10}H_9NO_2$	0.028	$M^{+\bullet *}$	0.0069	N.A	N.A	N.A	N.A	N.A	N.A	N.A	Indoleacetic acid	Indole
179.033885	179.033892	$C_9H_6O_4$	-0.041	$[M + H]^{+*}$, $M^{+\bullet}$	0.0421	0.0180	0.0291	0.0512	0.0148	0.0413	0.0099	0.0224	Aesculetin	Phenolics
179.070271	179.070269	$C_{10}H_{10}O_3$	0.010	$[M + H]^{+*}$, $M^{+\bullet}$	0.0469	0.0544	0.0496	0.0249	0.0539	0.2560	0.0413	0.0141	Dehydroconiferyl alcohol	Phenolics
179.106656	179.106668	$C_{11}H_{14}O_2$	-0.067	$[M + H]^{+*}$, $M^{+\bullet}$	0.0169	0.0101	0.0125	0.0122	0.0196	0.0301	0.0351	0.0141	Methyl eugenol	Terpenoid
180.078096	180.078102	$C_{10}H_{12}O_3$	-0.033	$[M + H]^{+}$, $M^{+\bullet *}$	0.0944	0.1060	0.2225	0.0225	0.0646	0.2816	0.0891	0.0252	Coniferyl alcohol	Phenolics
181.049535	181.049556	$C_9H_8O_4$	-0.117	$[M + H]^{+*}$, $M^{+\bullet}$	0.0294	0.0164	0.0276	0.0204	0.0082	0.0421	0.0178	0.0059	Caffeic acid	Phenolic acid
181.101177	181.101189	$C_{14}H_{12}$	-0.070	$[M + H]^{+*}$, $M^{+\bullet}$	0.0366	0.1120	N.A	0.1638	N.A	N.A	N.A	N.A	Stilbenes	Stilbenoid
182.093746	182.093747	$C_{10}H_{14}O_3$	-0.007	$[M + H]^{+}$, $M^{+\bullet *}$	0.1021	0.3079	0.0228	0.0288	0.0343	0.3640	0.0148	0.0104	Dihydroconiferyl alcohol	Phenolics
183.065185	183.065191	$C_9H_{10}O_4$	-0.032	$[M + H]^{+*}$, $M^{+\bullet}$	0.0248	N.A	0.0189	N.A	0.0268	0.0550	0.0229	0.0112	3-Hydroxy-3-(3-hydroxyphenyl) propionic acid	Phenolic acid
183.137956	183.137977	$C_{11}H_{18}O_2$	-0.114	$[M + H]^{+*}$, $M^{+\bullet}$	0.0100	0.0048	0.0041	0.0110	0.0080	0.0195	0.0247	0.0163	Terpinyl formate	Terpenoid
185.044450	185.044468	$C_8H_8O_5$	-0.101	$[M + H]^{+*}$, $M^{+\bullet}$	0.0096	0.0033	0.0053	0.0054	0.0058	0.0143	0.0000	0.0073	4-O-Methylgallic acid	Phenolic acid
185.153606	185.153616	$C_{11}H_{20}O_2$	-0.052	$[M + H]^{+}$	0.0127	0.0066	0.0042	0.0128	0.0092	0.0136	0.0426	0.0220	Methyl citronellate, z-3.Hexenyl-isovalerate	Terpenoid
193.085921	193.085944	$C_{11}H_{12}O_3$	-0.122	$[M + H]^{+}$, $M^{+\bullet *}$	0.0459	0.0175	0.0423	0.0232	0.0269	0.0678	0.0436	0.0107	p-Coumaric acid ethyl ester	Phenolic acid
194.057360	194.057384	$C_{10}H_{10}O_4$	-0.123	$[M + H]^{+}$, $M^{+\bullet *}$	0.1046	0.0937	0.1663	0.0842	0.0217	0.1497	0.0341	0.0300	Ferulic acid	Phenolic acid
195.137956	195.137957	$C_{12}H_{18}O_2$	-0.006	$[M + H]^{+*}$, $M^{+\bullet}$	0.0216	0.0072	0.0073	0.0094	0.0295	0.0294	0.0300	0.0153	Cis-carvyl acetate, Myrtenyl	Terpenoid
197.153606	197.153621	$C_{12}H_{20}O_2$	-0.072	$[M + H]^{+*}$, $M^{+\bullet}$	0.0101	0.0044	0.0035	0.0096	0.0088	0.0106	0.0278	0.0176	Terpinyl acetate, Bornyl acetate	Terpenoid
199.060100	199.060109	$C_9H_{10}O_5$	-0.048	$[M + H]^{+*}$, $M^{+\bullet}$	0.0111	0.0028	0.0060	0.0057	0.0055	0.0108	0.0064	N.A	Syringic acid	Phenolic acid
201.184906	201.184927	$C_{12}H_{24}O_2$	-0.103	$[M + H]^{+}$	N.A	N.A	N.A	N.A	N.A	N.A	0.0107	N.A	Lauric acid	Fatty acid
204.187252	204.187251	$C_{15}H_{24}$	0.008	$[M + H]^{+}$, $M^{+\bullet *}$	0.0230	0.0059	0.0026	0.0059	0.6991	0.4385	0.2227	0.1236	Sesquiterpene	Terpenoid
207.101571	207.101571	$C_{12}H_{14}O_3$	-0.001	$[M + H]^{+*}$, $M^{+\bullet}$	0.0336	0.0235	0.0250	0.0200	0.0253	0.0578	0.0439	0.0102	Acetyl eugenol	Terpenoid
207.137956	207.137969	$C_{13}H_{18}O_2$	-0.059	$[M + H]^{+*}$, $M^{+\bullet}$	0.0898	0.0324	0.1056	0.0203	0.0830	0.1425	0.0772	0.0238	Phenyl ethyl-3-methyl butanote	Terpenoid
207.174342	207.174314	$C_{14}H_{22}O$	0.135	$[M + H]^{+*}$, $M^{+\bullet}$	0.0102	0.0054	N.A	N.A	0.0468	0.0222	0.0286	0.0156	Alpha irone	Terpenoid

211.096485	211.096444	$C_{11}H_{14}O_4$	0.197	$[M + H]^{+*}$, $M^{+\bullet}$	0.0109	0.0037	0.0106	0.0054	0.0078	0.0115	0.0180	0.0064	Sinapyl alcohol	Phenolics
211.169256	211.169234	$C_{13}H_{22}O_2$	0.109	$[M + H]^{+*}$, $M^{+\bullet}$	0.0191	0.0094	0.0070	0.0081	0.0136	0.0167	0.0330	0.0174	Isobornyl propionate, 3-methyl butanoic acid, 2- methyl butyrate	Terpenoid
213.091006	213.091015	$C_{14}H_{12}O_2$	-0.041	$[M + H]^{+*}$, $M^{+\bullet}$	0.0368	0.0206	0.0084	0.0262	0.0368	0.0192	0.0309	0.0301	Pinosylvin	Stilbenoid
217.158692	217.158684	$C_{15}H_{20}O$	0.034	$[M + H]^{+*}$, $M^{+\bullet}$	0.0181	0.0059	0.0074	0.0063	0.0267	0.0251	0.0263	0.0155	Hexyl cinnamaldehyde	
219.174342	219.174320	$C_{15}H_{22}O$	0.099	$[M + H]^{+*}$, $M^{+\bullet}$	0.0459	0.0089	0.0213	0.0077	0.0805	0.0503	0.0420	0.0147	Nootkatone	Terpenoid
221.189992	221.189972	$C_{15}H_{24}O$	0.088	$[M + H]^{+*}$, $M^{+\bullet}$	0.0803	0.0196	0.0178	0.0102	0.1130	0.1213	0.0467	0.0164	Epi cubenol, caryophyllene oxide	Terpenoid
223.205642	223.205631	$C_{15}H_{26}O$	0.050	$[M + H]^{+*}$, $M^{+\bullet}$	0.0108	0.0040	0.0032	0.0080	0.0086	0.0096	0.0318	0.0149	Cadinol, fersonal, nerolidol	Terpenoid
225.075750	225.075740	$C_{11}H_{12}O_5$	0.043	$[M + H]^{+*}$, $M^{+\bullet}$	0.0168	0.0060	N.A	0.0046	N.A	N.A	N.A	N.A	Sinapinic acid	Phenolic acid
227.091400	227.091409	$C_{11}H_{14}O_5$	-0.040	$[M + H]^{+*}$, $M^{+\bullet}$	0.0139	0.0101	0.0110	N.A	0.0086	0.0170	0.0078	0.0075	Dihydrosinapic acid	Phenolic acid
227.106656	227.106638	$C_{15}H_{14}O_2$	0.079	$[M + H]^{+*}$, $M^{+\bullet}$	0.0222	0.0167	0.0097	0.0079	0.0603	0.0184	0.0179	0.0121	Pinosylvin monomethyl ether	Stilbenoid
229.085921	229.085900	$C_{14}H_{12}O_3$	0.093	$[M + H]^{+*}$, $M^{+\bullet}$	0.0584	0.0257	0.0148	0.0188	0.0699	0.1834	0.0137	0.0123	Resveratrol	Stilbenoid
229.216207	229.216201	$C_{14}H_{28}O_2$	0.023	$[M + H]^{+}$	0.0072	N.A	0.0027	N.A	0.0053	0.0066	0.0469	0.0150	Myristic acid	Fatty acid
231.101571	231.101553	$C_{14}H_{14}O_3$	0.078	$[M + H]^{+*}$, $M^{+\bullet}$	0.0361	0.0199	0.0141	0.0767	0.0215	0.0151	0.0242	0.0455	Dihydro-resveratrol	Stilbenoid
244.073010	244.072979	$C_{14}H_{12}O_4$	0.129	$[M + H]^{+}$, $M^{+\bullet *}$	0.0315	1.3089	0.0306	0.0207	0.0094	3.6935	0.0217	0.0161	Piceatannol	Stilbenoid
247.096485	247.096466	$C_{14}H_{14}O_4$	0.077	$[M + H]^{+*}$, $M^{+\bullet}$	0.0396	0.0252	0.0294	0.0967	0.0211	0.0190	0.0165	0.0239	Dihydropiceatannol	Stilbenoid
253.195077	253.195065	$C_{19}H_{24}$	0.050	$[M + H]^{+}$	0.0114	0.0117	0.0068	0.0063	0.1043	0.1917	0.1397	0.0746	18-Norabieta-4,6,8,11,13-pentaene, Simonellite	Terpenoid
255.231857	255.231832	$C_{16}H_{30}O_2$	0.097	$[M + H]^{+}$	0.0590	0.0185	0.0242	0.0367	0.0217	0.0465	0.2132	0.1123	Palmitoleic acid	Fatty acid
257.080835	257.080820	$C_{15}H_{12}O_4$	0.059	$[M + H]^{+*}$, $M^{+\bullet}$	0.0246	0.0571	0.0199	0.0047	0.0138	0.0685	0.0119	0.0079	Pinocembrin	Flavanone
257.226377	257.226382	$C_{19}H_{28}$	-0.019	$[M + H]^{+*}$, $M^{+\bullet}$	0.0216	0.0202	0.0335	0.0122	1.5266	0.4463	2.4721	0.1427	Norabietatriene	Terpenoid
257.247507	257.247495	$C_{16}H_{32}O_2$	0.047	$[M + H]^{+}$	0.1676	0.0141	0.0762	0.0133	0.0468	0.0757	1.2191	0.1715	Palmitic acid	Fatty acid
258.088660	258.088639	$C_{15}H_{14}O_4$	0.083	$[M + H]^{+}$, $M^{+\bullet *}$	0.0287	2.9650	0.0173	0.0134	0.0086	3.6121	0.0085	0.0121	Isorhapontigenin	Stilbenoid
271.060100	271.060087	$C_{15}H_{10}O_5$	0.047	$[M + H]^{+*}$, $M^{+\bullet}$	0.0669	0.1146	0.3315	0.5627	0.1118	0.3531	0.1380	0.5279	Apigenin	Flavone
271.205642	271.205626	$C_{19}H_{26}O$	0.059	$[M + H]^{+*}$, $M^{+\bullet}$	0.0321	0.0413	0.0340	0.0182	0.2968	0.4406	0.5382	0.2772	7-Oxo-19-norabieta-8,11,13-triene, Nordehydroabietal	Terpenoid
271.242027	271.242011	$C_{20}H_{30}$	0.061	$[M + H]^{+*}$, $M^{+\bullet}$	0.0115	0.0119	0.0119	0.0104	0.5509	0.4121	0.3513	0.1524	Abieta-8,11,13-triene, Dehydroabietane	Terpenoid
273.075750	273.075751	$C_{15}H_{12}O_5$	-0.003	$[M + H]^{+*}$, $M^{+\bullet}$	0.0796	0.0743	0.3987	0.0697	0.1115	0.2060	0.2559	0.0560	Naringenin	Flavanone
273.096879	273.096864	$C_{12}H_{16}O_7$	0.058	$[M + H]^{+*}$, $M^{+\bullet}$	0.0140	N.A	0.0132	N.A	N.A	N.A	N.A	N.A	Arbutin	Glucoside
273.257677	273.257665	$C_{20}H_{32}$	0.047	$[M + H]^{+*}$, $M^{+\bullet}$	0.0241	0.0166	0.0087	0.0141	15.2740	0.4366	0.2054	0.1109	Diterpene	Terpenoid
275.091400	275.091406	$C_{15}H_{14}O_5$	-0.023	$[M + H]^{+*}$, $M^{+\bullet}$	0.0382	0.0293	0.0935	0.0226	0.0155	0.0885	0.0713	0.0886	Epiafzelechin	Flavan-3-ol
279.231857	279.231850	$C_{18}H_{30}O_2$	0.024	$[M + H]^{+*}$, $M^{+\bullet}$	0.0425	0.0427	0.0403	0.1008	0.1394	0.1765	0.5524	0.3758	Pinolenic, linolenic	Fatty acid
281.247507	281.247459	$C_{18}H_{32}O_2$	0.172	$[M + H]^{+*}$, $M^{+\bullet}$	0.0457	0.0318	0.0387	0.0930	0.0181	0.0571	0.3837	0.2704	Linoleic acid	Fatty acid

283.263157	283.263167	$C_{18}H_{34}O_2$	-0.037	$[M + H]^{+*}$, $M^{+\bullet}$	0.0876	0.0634	0.0511	0.1159	0.0273	0.0579	0.7193	0.4546	Oleic acid	Fatty acid
285.221292	285.221278	$C_{20}H_{28}O$	0.049	$[M + H]^{+*}$, $M^{+\bullet}$	0.0271	0.0205	0.0521	0.0167	0.9460	0.3975	1.5544	0.1239	Dehydroabietal, Dehydroferruginol	Terpenoid
285.278807	285.278785	$C_{18}H_{36}O_2$	0.077	$[M + H]^{+}$	0.3233	0.0395	0.2132	0.0469	0.1392	0.1706	4.2272	0.4553	Stearic acid	Fatty acid
286.047189	286.047202	$C_{15}H_{10}O_6$	-0.045	$[M + H]^{+}$, $M^{+\bullet *}$	1.1019	0.8147	1.1934	23.1397	0.8661	2.9902	0.2991	32.8414	Kaempferol	Flavonol
286.083575	286.083573	$C_{16}H_{14}O_5$	0.007	$[M + H]^{+}$, $M^{+\bullet *}$	0.0883	0.1767	0.1449	0.0765	0.0326	0.1584	0.0445	0.0619	Dihydrobiochanin A	Flavanone
287.127786	287.127775	$C_{17}H_{18}O_4$	0.038	$[M + H]^{+*}$, $M^{+\bullet}$	0.1163	0.1633	0.0696	0.0476	0.0622	0.1506	0.0787	0.0461	4'-Hydroxy-3,4,5-trimethoxystilbene	Stilbenoid
287.236942	287.236963	$C_{20}H_{30}O$	-0.074	$[M + H]^{+*}$, $M^{+\bullet}$	0.0313	0.0197	0.0341	0.0186	0.7952	0.2677	0.9376	0.0830	Ferruginol	Terpenoid
288.062840	288.062866	$C_{15}H_{12}O_6$	-0.091	$[M + H]^{+}$, $M^{+\bullet *}$	0.4386	1.8695	1.6528	2.4474	0.2005	1.6308	0.1416	1.3157	Aromadendrin	Flavanonol
289.252592	289.252610	$C_{20}H_{32}O$	-0.062	$[M + H]^{+*}$, $M^{+\bullet}$	0.0215	0.0217	0.0378	0.0279	0.4620	0.2369	0.4396	0.0800	Isopimarol, Abietol	Terpenoid
290.078490	290.078480	$C_{15}H_{14}O_6$	0.033	$[M + H]^{+}$, $M^{+\bullet *}$	1.8804	6.9504	17.8318	8.8678	0.9928	4.8593	4.7457	3.7962	Catechin	Flavan-3-ol
291.231857	291.231880	$C_{19}H_{30}O_2$	-0.081	$[M + H]^{+}$	0.0544	0.0283	0.0367	0.0492	2.3862	0.0526	0.2300	0.0980	19- Noragathic acid	Terpenoid
291.268242	291.268253	$C_{20}H_{34}O$	-0.035	$[M + H]^{+*}$, $M^{+\bullet}$	0.0242	0.0365	0.0333	0.0627	0.1170	0.0426	0.1349	0.0950	Isoabienal, Anticopalol	Terpenoid
292.239682	292.239687	$C_{19}H_{32}O_2$	-0.019	$M^{+\bullet *}$	N.A	N.A	N.A	0.0107		0.0218	0.0979	0.0501	Pinolenic methyl ester, 15-norpinifolic acid	Terpenoid
293.247507	293.247509	$C_{19}H_{32}O_2$	-0.007	$[M + H]^{+}$	0.0184	0.0185	0.0230	0.0611	0.0000	0.0644	0.2779	0.1056	Pinolenic methyl ester, 15-norpinifolic acid	Terpenoid
297.315192	297.315179	$C_{20}H_{40}O$	0.042	$[M + H]^{+}$	0.0065	0.0033	0.0039	0.0118	N.A	N.A	0.0648	N.A	Phytol	Terpenoid
299.112529	299.112497	$C_{14}H_{18}O_7$	0.107	$[M + H]^{+*}$, $M^{+\bullet}$	0.0186	0.0091	0.0192	0.0109	0.0073	0.0438	0.0138		Picein	Phenolic glycoside
299.127786	299.127808	$C_{18}H_{18}O_4$	-0.075	$[M + H]^{+*}$, $M^{+\bullet}$	2.2970	0.5073	0.7610	0.1203	1.9429	0.7541	0.2708	0.0575	Ligballinol (p-coumarylresinol)	Lignan
299.200557	299.200583	$C_{20}H_{26}O_2$	-0.087	$[M + H]^{+*}$, $M^{+\bullet}$	0.0978	0.0753	0.1124	0.0434	0.6927	0.4948	1.2832	0.2791	Abieta-6,8,11,13-tetraen-18-oic acid	Terpenoid
300.062840	300.062840	$C_{16}H_{12}O_6$	0.000	$[M + H]^{+}$, $M^{+\bullet *}$	0.6927	0.5187	0.3437	0.1846	0.2825	0.3649	0.0691	0.2022	Hispidulin	Flavone
301.091794	301.091799	$C_{13}H_{16}O_8$	-0.018	$[M + H]^{+*}$, $M^{+\bullet}$	0.0532	0.0155	0.0381	0.0235	0.0239	0.0285	0.0081	0.0149	4-Hydroxybenzoic acid 4-O-glucoside	Phenolic glycoside
301.179821	301.179845	$C_{19}H_{24}O_3$	-0.081	$[M + H]^{+*}$, $M^{+\bullet}$	0.1122	0.1309	0.0784	0.0816	N.A	0.1425	0.4452	0.1596	Z-bornyl-p-coumarate	Phenolics
301.216207	301.216217	$C_{20}H_{28}O_2$	-0.035	$[M + H]^{+*}$, $M^{+\bullet}$	0.0997	0.0657	0.1878	0.0612	2.0002	0.6026	2.6539	0.4684	Dehydroabietic acid	Terpenoid
302.042104	302.042121	$C_{15}H_{10}O_7$	-0.057	$[M + H]^{+}$, $M^{+\bullet *}$	1.3398	0.1018	2.7107	1.8616	0.6897	0.4761	0.0437	1.8939	Quercetin	Flavonol
302.224032	302.224035	$C_{20}H_{30}O_2$	-0.012	$[M + H]^{+}$, $M^{+\bullet *}$	0.3169	0.6485	0.5622	0.6177	11.4255	3.8434	24.0070	5.5183	Abietic acid	Terpenoid
303.086315	303.086310	$C_{16}H_{14}O_6$	0.016	$[M + H]^{+*}$, $M^{+\bullet}$	0.2044	0.1748	1.0969	0.1152	0.1164	0.3214	0.1044	0.0539	Methylaromadendrin	Flavanonol
303.107444	303.107476	$C_{13}H_{18}O_8$	-0.106	$[M + H]^{+*}$, $M^{+\bullet}$	0.0127	0.0037	0.0236	0.0077	0.0053		0.0091	N.A	Tachioside	Phenolic glycoside
305.065579	305.065581	$C_{15}H_{12}O_7$	-0.004	$[M + H]^{+*}$, $M^{+\bullet}$	0.1694	0.0751	0.1964	0.4417	0.1713	0.1602	0.0381	0.1333	Taxifolin	Flavanonol
305.101965	305.101925	$C_{16}H_{16}O_6$	0.130	$[M + H]^{+*}$, $M^{+\bullet}$	0.0593	0.0321	0.1373	0.0260	0.0330	0.0719	0.0668	0.0100	3'-O-methylcatechin	Flavan-3-ol
305.247507	305.247508	$C_{20}H_{32}O_2$	-0.005	$[M + H]^{+*}$, $M^{+\bullet}$	0.0680	0.0483	0.0953	0.0523	1.1240	0.1034	0.3904	0.0964	Anticopalic acid	Terpenoid
306.073404	306.073426	$C_{15}H_{14}O_7$	-0.069	[M + H]+, $M^{+\bullet *}$	1.4213	1.5443	1.7841	7.5764	0.6527	1.5087	0.0317	3.0854	Gallocatechol	Flavan-3-ol

307.263157	307.263155	$C_{20}H_{34}O_2$	0.006	$[M + H]^+$	0.0396	0.0520	0.0965	0.0737	0.2080	0.0818	0.2202	0.1585	18-Hydroxymanool	Terpenoid
309.242421	309.242461	$C_{19}H_{32}O_3$	-0.127	$[M + H]^{+*}$, $M^{+\bullet}$	0.0247	0.0305	0.0256	0.0723	0.0432	0.0368	0.1554	0.2156	Nor-16-imbricatolic acid	Terpenoid
314.078490	314.078522	$C_{17}H_{14}O_6$	-0.104	$[M + H]^+$, $M^{+\bullet *}$	0.3122	0.5957	1.1548	1.2376	0.1293	0.3304	0.1353	0.7100	3-O-Acetylpinobanksin	
314.224032	314.224022	$C_{21}H_{30}O_2$	0.029	$[M + H]^+$, $M^{+\bullet *}$	0.0133	0.0171	0.0059	0.0159	0.5178	0.0776	0.0821	0.1490	Methyl Dehydroabietate	Terpenoid
315.195471	315.195428	$C_{20}H_{26}O_3$	0.138	$[M + H]^{+*}$, $M^{+\bullet}$	0.1671	0.1232	0.1015	0.1008	0.2719	0.2681	0.7014	0.2810	Xanthoperol,7-Oxodehydroabietic acid	Terpenoid
316.057754	316.057767	$C_{16}H_{12}O_7$	-0.042	$[M + H]^+$, $M^{+\bullet *}$	1.9601	0.4224	0.8228	2.3542	1.2959	1.1619	0.0512	2.2569	Isorhamnetin	Flavonol
316.239682	316.239640	$C_{21}H_{32}O_2$	0.133	$[M + H]^+$, $M^{+\bullet *}$	0.0127	0.0378	0.0050	0.0312	0.2686	0.2044	0.4340	0.8264	Methyl Abietate	Terpenoid
317.086709	317.086735	$C_{13}H_{16}O_9$	-0.085	$[M + H]^{+*}$, $M^{+\bullet}$	N.A	0.0053	0.0139	0.0243	N.A	N.A	N.A	N.A	Protocatechuic acid 4-O-glucoside	Phenolic glycoside
317.123094	317.123119	$C_{14}H_{20}O_8$	-0.080	$[M + H]^{+*}$, $M^{+\bullet}$	0.0157	N.A	N.A	N.A	N.A	N.A	N.A	N.A	3,4-Dimethoxyphenyl-beta-D-glucopyranoside	Phenolic glycoside
317.174736	317.174695	$C_{19}H_{24}O_4$	0.128	$[M + H]^{+*}$, $M^{+\bullet}$	0.0513	0.0484	0.0395	0.0341	0.0343	0.0358	0.1636	0.0860	Gibberellin A9	Terpenoid
317.211121	317.211134	$C_{20}H_{28}O_3$	-0.040	$[M + H]^+$	0.0935	0.0784	0.1370	0.0495	0.3617	0.3918	0.6031	0.2576	12-Hydroxydehydroabietic Acid	Terpenoid
318.037019	318.037047	$C_{15}H_{10}O_8$	-0.088	$[M + H]^+$, $M^{+\bullet *}$	0.0529	0.0174	0.0329	0.5802	0.0850	0.4891	0.0000	0.8264	Myricetin	Flavonoid
319.081229	319.081216	$C_{16}H_{14}O_7$	0.043	$[M + H]^{+*}$, $M^{+\bullet}$	0.1296	0.0662	0.2501	0.0716	0.0522	0.1270	0.0183	0.0371	Cedeodarin	Phenolics
319.226771	319.226788	$C_{20}H_{30}O_3$	-0.051	$[M + H]^{+*}$, $M^{+\bullet}$	0.0543	0.0357	0.0772	0.0390	0.1856	0.0739	0.3465	0.1302	3-oxoanticopalic Acid	Terpenoid
321.060494	321.060484	$C_{15}H_{12}O_8$	0.030	$[M + H]^{+*}$, $M^{+\bullet}$	0.0360	0.0130	0.0252	0.1567	0.0800	0.1502	0.0000	0.0549	Ampelopsin	Flavanonol
321.096879	321.096923	$C_{16}H_{16}O_7$	-0.135	$[M + H]^{+*}$, $M^{+\bullet}$	0.0537	0.0350	0.0830	0.0156	0.0396	0.3133	0.0240	0.0131	4’-O-Methylepigallocatechin	Flavan-3-ol
321.242421	321.242388	$C_{20}H_{32}O_3$	0.104	$[M + H]^{+*}$, $M^{+\bullet}$	0.0370	0.0286	0.0518	0.0342	0.0915	0.0420	0.1580	0.0680	Isocupressic, Hydroxyanticopalic Agatholic acid, Imbricataloic acid	Terpenoid
323.258071	323.258074	$C_{20}H_{34}O_3$	-0.007	$[M + H]^{+*}$, $M^{+\bullet}$	0.0324	0.0292	0.0530	0.0565	0.0335	0.0306	0.1050	0.0730	Imbricatolic, Dihydroagatholic acid	Terpenoid
325.091794	325.091754	$C_{15}H_{16}O_8$	0.122	$[M + H]^{+*}$, $M^{+\bullet}$	0.0139	0.0141	0.0830	0.0124	0.0057	0.0474	0.0530	0.0089	Skimmin	Phenolic glycoside
326.099619	326.099615	$C_{15}H_{18}O_8$	0.011	$[M + H]^{+*}$, $M^{+\bullet}$	0.0252	0.0119	0.0218	0.0572	0.0032	0.0054	0.0000	0.0000	p-Coumaric acid glucoside	Phenolic glycoside
327.159086	327.159112	$C_{20}H_{22}O_4$	-0.081	$[M + H]^{+*}$, $M^{+\bullet}$	0.9232	0.3183	1.6168	0.0742	0.7342	0.7362	0.3602	0.0299	Picealactone B	
328.130525	328.130537	$C_{19}H_{20}O_5$	-0.036	$[M + H]^+$, $M^{+\bullet *}$	33.0613	17.8940	8.0205	3.4426	6.0870	4.0908	1.7332	1.2619	Demethoxylpinoresinol	Lignan
329.211121	329.211111	$C_{21}H_{28}O_3$	0.033	$[M + H]^{+*}$, $M^{+\bullet}$	0.0306	0.0216	0.0149	0.0192	0.0936	0.0776	0.0837	0.1251	7-Methyl-oxodehydroabietate	Terpenoid
330.073404	330.073405	$C_{17}H_{14}O_7$	-0.003	$[M + H]^+$, $M^{+\bullet *}$	0.6418	0.6806	1.3555	0.3327	0.4982	0.7101	0.1518	0.2307	Quercetin 3,4'-dimethyl ether	Flavonol
330.146175	330.146129	$C_{19}H_{22}O_5$	0.141	$[M + H]^+$, $M^{+\bullet *}$	1.0862	2.2233	1.2076	0.3943	0.1901	0.6295	0.3133	0.1665	Gibberellin A7	Terpenoid
331.081229	331.081198	$C_{17}H_{14}O_7$	0.095	$[M + H]^+$	0.0944	N.A	N.A	0.0777	0.1472	0.1863	0.0421	N.A	Quercetin 3,4'-dimethyl ether	Flavonol
331.190386	331.190412	$C_{20}H_{26}O_4$	-0.078	$[M + H]^+$	0.0957	0.0538	0.0840	0.0507	0.0722	0.0673	0.2094	0.0588	15-Hydroxy-7-oxodehydroabietic acid	Terpenoid
332.052669	332.052697	$C_{16}H_{12}O_8$	-0.085	$[M + H]^+$, $M^{+\bullet *}$	0.3979	0.0490	0.1854	1.0412	0.1675	0.0765	0.0000	0.8627	Laricitrin	Flavonol
332.198211	332.198216	$C_{20}H_{28}O_4$	-0.015	$[M + H]^+$, $M^{+\bullet *}$	0.0868	0.0923	0.1077	0.0580	0.0318	0.0655	0.1478	0.0551	Pinusolic acid, Gibberellin A12	Terpenoid

333.169650	333.169605	$C_{19}H_{24}O_5$	0.137	$[M + H]^{+*}$, $M^{+\bullet}$	0.0330	0.0261	0.0322	0.0232	0.0128	0.0174	0.0593	0.0223	Gibberellin A20	Terpenoid
333.278807	333.278828	$C_{22}H_{36}O_2$	-0.062	$[M + H]^{+*}$, $M^{+\bullet}$	0.0138	0.0158	0.0081	0.0436	0.0148	0.0215	0.0572	0.0504	Acetyldihydroabietol	Terpenoid
335.221686	335.221696	$C_{20}H_{30}O_4$	-0.031	$[M + H]^{+*}$, $M^{+\bullet}$	0.0583	0.0378	0.0743	0.0333	0.2720	0.1435	0.1450	0.0938	Dehydropinifolic acid	Terpenoid
335.258071	335.258109	$C_{21}H_{34}O_3$	-0.113	$[M + H]^{+*}$, $M^{+\bullet}$	0.0271	0.0347	0.0289	0.1140	5.7854	1.7415	2.7076	4.1066	Methyl ester of isocupressic	Terpenoid
337.237336	337.237339	$C_{20}H_{32}O_4$	-0.009	$[M + H]^{+*}$, $M^{+\bullet}$	0.0223	0.0242	0.0310	0.0257	0.0416	0.0186	0.0693	0.0447	Pinifolic acid	Terpenoid
339.107444	339.107392	$C_{16}H_{18}O_8$	0.154	$[M + H]^{+*}$, $M^{+\bullet}$	0.0541	0.0246	0.1052	0.0127	0.0774	0.1232	0.0188	0.0106	3-p-Coumaroylquinic acid	Phenolic acid
341.138350	341.138340	$C_{20}H_{20}O_5$	0.029	$[M + H]^{+*}$, $M^{+\bullet}$	10.5325	N.A	1.2526	0.3539	2.8085	1.4094	0.4444	0.1054	8-Prenylnaringenin	Flavonoid
342.130919	342.130964	$C_{16}H_{22}O_8$	-0.132	$[M + H]^{+}$, $M^{+\bullet *}$	0.1653	0.2866	2.2654	0.0142	0.0584	0.1619	0.5487	0.0336	Coniferin	Phenolic glycoside
343.102359	343.102394	$C_{15}H_{18}O_9$	-0.104	$[M + H]^{+*}$, $M^{+\bullet}$	N.A	N.A	0.0314	N.A	N.A	N.A	N.A	N.A	Glucocaffeic acid	Phenolic glycoside
344.146569	344.146609	$C_{16}H_{24}O_8$	-0.115	$[M + H]^{+}$, $M^{+\bullet *}$	0.1792	0.4531	0.2067	0.0376	0.0190	0.1596	0.0378	0.0097	Dihydroconiferin	Phenolic glycoside
345.096879	345.096868	$C_{18}H_{16}O_7$	0.034	$[M + H]^{+*}$, $M^{+\bullet}$	0.0841	0.0630	0.1499	0.0320	0.0459	0.1203	0.0357	0.0254	Cirsilineol	Flavone
346.068319	346.068312	$C_{17}H_{14}O_8$	0.020	$[M + H]^{+*}$, $M^{+\bullet}$	0.6557	0.1534	0.5538	1.6253	0.2565	0.1238	0.0227	1.1918	Syringetin	Flavonol
346.141090	346.141055	$C_{19}H_{22}O_6$	0.101	$[M + H]^{+}$, $M^{+\bullet *}$	11.9544	9.0958	6.1776	1.0196	2.0334	2.9284	1.7660	0.3871	Taxiresinol, Gibberellin A3	Lignan
347.221686	347.221632	$C_{21}H_{30}O_4$	0.154	$[M + H]^{+*}$, $M^{+\bullet}$	0.0254	0.0124	0.0163	0.0204	0.0610	0.0350	0.0641	0.1000	Pinusolide	Terpenoid
349.164565	349.164559	$C_{19}H_{24}O_6$	0.017	$[M + H]^{+*}$, $M^{+\bullet}$	0.0194	0.0109	0.0209	0.0119	0.0093	0.0112	0.0165	0.0094	Gibberellin A1	Terpenoid
349.200950	349.200984	$C_{20}H_{28}O_5$	-0.097	$[M + H]^{+*}$, $M^{+\bullet}$	0.0764	0.0432	0.0557	0.0360	0.0302	0.0417	0.1189	0.0363	Gibberellin A15	Terpenoid
351.216601	351.216595	$C_{20}H_{30}O_5$	0.016	$[M + H]^{+*}$, $M^{+\bullet}$	0.0371	0.0296	0.0625	0.0246	0.0344	0.0291	0.0626	0.0474	Andrographolide	
351.252986	351.253020	$C_{21}H_{34}O_4$	-0.096	$[M + H]^{+*}$, $M^{+\bullet}$	0.0402	0.0340	0.0214	0.0731	1.0833	0.1619	0.3208	0.7838	Pinifolate, Nor-16-acetylimbricatolic	Terpenoid
354.094534	354.094518	$C_{16}H_{18}O_9$	0.044	$[M + H]^{+}$, $M^{+\bullet *}$	0.0166	0.0250	0.0158	0.0000	0.0091	0.0217	0.0211	0.0075	Chlorogenic Acid	Phenolic acid
355.117615	355.117607	$C_{20}H_{18}O_6$	0.022	$[M + H]^{+*}$, $M^{+\bullet}$	0.1410	0.1488	0.1491	0.0000	0.0721	0.1472	0.0522	0.0244	Episesamin	
357.081623	357.081641	$C_{15}H_{16}O_{10}$	-0.050	$[M + H]^{+}$	0.0048	N.A	N.A	N.A	N.A	N.A	N.A	N.A	Caffeic acid 3-O-glucuronide	Phenolic glycoside
357.118009	357.118012	$C_{16}H_{20}O_9$	-0.010	$[M + H]^{+}$	0.0304	N.A	0.0403	0.0066	0.0078	0.0054	0.0065	N.A	1-O-Feruloyl-beta-D-glucose	Phenolic glycoside
357.133265	357.133277	$C_{20}H_{20}O_6$	-0.034	$[M + H]^{+*}$, $M^{+\bullet}$	0.3173	0.2402	0.3460	0.0580	0.1268	0.3011	0.1420	0.0274	α-Conidendrin	Lignan
358.125834	358.125843	$C_{16}H_{22}O_9$	-0.026	$M^{+\bullet *}$	0.3498	0.0879	0.5577	0.0569	0.0116	0.0195	0.0228	0.0076	Junipediol B 8-glucoside	Flavonoid glucoside
358.141090	358.141114	$C_{20}H_{22}O_6$	-0.067	$[M + H]^{+}$, $M^{+\bullet *}$	0.7686	1.7583	2.0595	0.2437	0.1255	0.6930	0.5539	0.0889	Pinoresinol	Lignan
360.083969	360.083958	$C_{18}H_{16}O_8$	0.031	$[M + H]^{+}$, $M^{+\bullet *}$	0.0947	0.0382	0.0715	0.0491	0.0203	0.0321	0.0110	0.0448	Irigenin	Flavone
360.141484	360.141463	$C_{16}H_{24}O_9$	0.057	$[M + H]^{+}$, $M^{+\bullet *}$	0.0417	0.0382	1.2886	0.0061	0.0061	0.0169	0.2765	N.A	Junipediol A 8-glucoside	Flavonoid glucoside
360.156740	360.156751	$C_{20}H_{24}O_6$	-0.031	$[M + H]^{+}$, $M^{+\bullet *}$	2.8340	3.9036	1.5499	0.6984	0.7485	1.8734	0.4348	0.2814	Lariciresinol	Lignan
361.237336	361.237360	$C_{22}H_{32}O_4$	-0.066	$[M + H]^{+*}$, $M^{+\bullet}$	0.0082	0.0065	0.0084	0.0124	0.0172	0.0183	0.0385	0.0886	7-Acetoxyabietic acid	Terpenoid
362.172390	362.172395	$C_{20}H_{26}O_6$	-0.013	$[M + H]^{+}$, $M^{+\bullet *}$	0.3701	0.4802	0.5255	0.1417	0.0594	0.1779	0.0730	0.0264	Secoisolariciresinol	Lignan
363.252986	363.253010	$C_{22}H_{34}O_4$	-0.067	$[M + H]^{+*}$, $M^{+\bullet}$	0.0062	0.0074	0.0055	0.0196	0.0396	0.0196	0.0439	0.0808	3 beta- Acetoxyanticopalic, Acetylisocupressic	Terpenoid

364.151655	364.151670	$C_{19}H_{24}O_7$	-0.042	$[M + H]^{+}$, $M^{+\bullet}*$	0.0139	0.0179	0.0175	0.0054	0.0000	0.0112	0.0088	N.A	Gibberellin A8	Terpenoid
365.268636	365.268648	$C_{22}H_{36}O_4$	-0.032	$[M + H]^{+}*$, $M^{+\bullet}$	0.0077	0.0169	N.A	0.0144	N.A	0.0153	0.0368	0.0483	Acetylimbricatolic acid	Terpenoid
368.110184	368.110204	$C_{17}H_{20}O_9$	-0.057	$M^{+\bullet}*$	0.071	0.1846	0.0596	0.0427	0.0054	0.1218	0.0000	0.0135	3-Feruloylquinic acid	Phenolic acid
371.097273	371.097301	$C_{16}H_{18}O_{10}$	-0.074	$[M + H]^{+}*$, $M^{+\bullet}$	N.A	N.A	0.0058	N.A	N.A	N.A	N.A	N.A	Ferulic acid 4-O-glucuronide	Phenolic glycoside
373.112923	373.112917	$C_{16}H_{20}O_{10}$	0.016	$[M + H]^{+}*$, $M^{+\bullet}$	N.A	N.A	0.0069	N.A	N.A	N.A	N.A	N.A	Dihydroferulic acid 4-O-glucuronide	Phenolic glycoside
373.149309	373.149248	$C_{17}H_{24}O_9$	0.162	$[M + H]^{+}*$, $M^{+\bullet}$	0.0084	N.A	N.A	N.A	N.A	N.A	N.A	N.A	Syringin	Phenolic glycoside
373.164565	373.164598	$C_{21}H_{24}O_6$	-0.087	$[M + H]^{+}*$, $M^{+\bullet}$	0.0169	0.0142	0.0481	0.0073	0.0106	0.0151	0.0255	N.A	Arctigenin	
374.099619	374.099628	$C_{19}H_{18}O_8$	-0.024	$[M + H]^{+}$, $M^{+\bullet}*$	0.0535	0.0297	0.0990	0.0246	0.0388	0.0418	0.0091	0.0838	Junipegenin C	Flavone
375.143829	375.143820	$C_{20}H_{22}O_7$	0.026	$[M + H]^{+}*$, $M^{+\bullet}$	0.1129	N.A	0.1045	0.0172	0.0633	0.2705	0.0268	0.0085	Hydroxymatairesinol, nortrachelogenin	Lignan
377.159480	377.159546	$C_{20}H_{24}O_7$	-0.177	$[M + H]^{+}*$, $M^{+\bullet}$	0.0164	0.0090	0.0176	0.0043	0.0120	0.0202	0.0118	0.0107	Todolactol A	Lignan
386.099619	386.099615	$C_{20}H_{18}O_8$	0.010	$[M + H]^{+}$, $M^{+\bullet}*$	0.0811	0.0418	0.3666	0.0635	0.0145	0.0347	0.0109	0.0264	Diferulic acid	Phenolic acid
386.157134	386.157163	$C_{18}H_{26}O_9$	-0.075	$[M + H]^{+}$, $M^{+\bullet}*$	0.0148	0.0122	1.7455	0.0000	0.0039	0.0000	1.0732	0.0000	Juniperoside	Phenolic glycoside
387.201344	387.201356	$C_{19}H_{30}O_8$	-0.029	$[M + H]^{+}*$, $M^{+\bullet}$	0.0120	0.0084	0.1098	0.0064	0.0067	0.0150	0.0217		Junipeionoloside, Corchoionoside C	
389.159480	389.159502	$C_{21}H_{24}O_7$	-0.057	$[M + H]^{+}*$, $M^{+\bullet}$	0.0509	0.0203	0.1044	0.0055	0.0257	0.0225	0.0415	N.A	Dihydrosamidin	
390.130919	390.130947	$C_{20}H_{22}O_8$	-0.071	$[M + H]^{+}$, $M^{+\bullet}*$	0.1597	0.7013	0.1452	0.0253	0.0271	0.4285	0.0178	0.0099	Piceid	Stilbenoid
390.152048	390.152033	$C_{17}H_{26}O_{10}$	0.039	$M^{+\bullet}*$	0.0242	0.0466	0.5828	0.0067	0.0023	0.0131	0.1012	N.A	Loganin	
391.175130	391.175062	$C_{21}H_{26}O_7$	0.174	$[M + H]^{+}*$, $M^{+\bullet}$	0.0120	0.0062	0.0152	0.0062	0.0039	N.A	0.0080	N.A	5'-Methoxylariciresinol	Lignan
397.382878	397.382864	$C_{29}H_{48}$	0.036	$[M + H]^{+}*$, $M^{+\bullet}$	0.0498	0.0391	0.0246	0.0434	3.0890	1.6878	1.2654	0.9491	Stigmastan-3,5-diene	Sterol
398.136004	398.136046	$C_{22}H_{22}O_7$	-0.105	$[M + H]^{+}$, $M^{+\bullet}*$	0.0965	0.0697	1.0248	0.0236	0.0238	0.0310	1.8971	0.0103	Deoxypodophyllotoxin	Lignan
399.128573	399.128576	$C_{18}H_{22}O_{10}$	-0.007	$[M + H]^{+}*$, $M^{+\bullet}$	N.A	0.0057	N.A	N.A	N.A	N.A	N.A	N.A	3-Sinapoylquinic acid	Phenolic acid
400.151655	400.151630	$C_{22}H_{24}O_7$	0.061	$[M + H]_{+}$, $M_{+\bullet}*$	0.0657	0.1459	0.6889	0.0238	0.0128	0.0669	0.4556	0.0144	Deoxypodorhizone	
401.232251	401.232164	$C_{24}H_{32}O_5$	0.215	$[M + H]_{+}*$, $M_{+\bullet}$	0.0066	0.0079	0.0042	0.0072	0.0098	0.0492	N.A	0.0215	Succinyl 7-oxodehydroabietol	Terpenoid
401.377793	401.377739	$C_{28}H_{48}O$	0.132	$[M + H]_{+}*$, $M_{+\bullet}$	N.A	0.0045	N.A	0.0157	0.0038	0.0108	0.0127	0.0249	Campesterol	Sterol
403.393443	403.393416	$C_{28}H_{50}O$	0.065	$[M + H]_{+}*$, $M_{+\bullet}$	N.A	0.0055	N.A	0.0560	0.0025	0.0115	0.0127	0.0755	Campestanol	Sterol
406.125834	406.125863	$C_{20}H_{22}O_9$	-0.072	$[M + H]_{+}$, $M_{+\bullet}*$	0.0209	1.7241	0.0277	0.0148	0.0051	1.9581	0.0000	0.0062	Astringin	Stilbenoid
413.123094	413.123140	$C_{22}H_{20}O_8$	-0.110	$[M + H]_{+}*$, $M_{+\bullet}$	0.0157	0.0208	0.0436	N.A	0.0106	0.0200	N.A	N.A	Podophyllotoxone	
413.377793	413.377832	$C_{29}H_{48}O$	-0.097	$[M + H]_{+}*$, $M_{+\bullet}$	0.0860	0.0574	0.0385	0.0415	0.2188	0.3141	0.3081	0.2479	Stigmasterol	Sterol
414.203690	414.203643	$C_{24}H_{30}O_6$	0.114	$[M + H]_{+}$, $M_{+\bullet}*$	0.0350	0.9153	0.0495	0.9114	0.0132	0.0523	0.0175	0.0282	Magnoshinin	Lignan
415.138744	415.138698	$C_{22}H_{22}O_8$	0.111	$[M + H]_{+}*$, $M_{+\bullet}$	0.0131	0.0097	0.0452	N.A	0.0104	0.0126	0.0176	N.A	Picropodophyllotoxin, Podophyllotoxin	
415.393443	415.393396	$C_{29}H_{50}O$	0.112	$[M + H]_{+}*$, $M_{+\bullet}$	N.A	0.0043	N.A	0.0171	0.0104	N.A	0.0234	0.0232	Beta-Sitosterol	Sterol
417.118009	417.117984	$C_{21}H_{20}O_9$	0.060	$[M + H]_{+}*$, $M_{+\bullet}$	N.A	N.A	N.A	0.1259	0.0282	0.0474	0.0114	0.0402	Apigenin 7-rhamnoside	Flavonoid

417.409093	417.409073	$C_{29}H_{52}O$	0.048	$[M + H]_{+}$*, $M_{+\bullet}$	N.A	0.0045	N.A	0.0360	0.0060	0.0091	0.0217	0.0355	Stigmastanol	Sterol
418.162219	418.162201	$C_{22}H_{26}O_8$	0.044	$[M + H]_{+}$*, $M_{+\bullet}$	0.0341	0.0579	1.2513	N.A	0.0047	0.0285	0.1688	N.A	Syringaresinol	Lignan
419.097273	419.097297	$C_{20}H_{18}O_{10}$	-0.057	$[M + H]_{+}$*, $M_{+\bullet}$	0.0047	N.A	0.0448	N.A	N.A	0.0072	0.0148	N.A	Salvianolic acid G	Phenolic acid
420.141484	420.141543	$C_{21}H_{24}O_9$	-0.141	$[M + H]_{+}$, $M_{+\bullet}$*	0.0412	16.5703	N.A	0.0113	0.0099	6.5395	0.0090	N.A	Isorhapontin	Stilbenoid
421.258465	421.258527	$C_{24}H_{36}O_6$	-0.146	$[M + H]_{+}$*, $M_{+\bullet}$	0.0059	0.0063	0.0038	0.0061	0.0106	0.0169	0.0184	0.0419	Succinylisocupresic, succinylagatholic	Terpenoid
421.440393	421.440369	$C_{29}H_{56}O$	0.057	$[M + H]_{+}$	0.0369	0.0304	0.0224	0.3997	0.0985	0.1084	0.3826	0.8514	9-nonacosene-2-one	
423.456043	423.456094	$C_{29}H_{58}O$	-0.120	$[M + H]_{+}$	0.0661	0.0493	0.0384	0.5786	0.1178	0.1839	0.4199	0.8777	10-nonacosanal, 10-nonacosanol	
427.393443	427.393489	$C_{30}H_{50}O$	-0.108	$[M + H]_{+}$*, $M_{+\bullet}$	N.A	0.0032	N.A	0.0104	N.A	N.A	N.A	N.A	Cycloartenol	Sterol
430.380532	430.380554	$C_{29}H_{50}O_2$	-0.050	$[M + H]_{+}$, $M_{+\bullet}$*	0.0132	0.1211	0.0090	0.0239	1.1481	0.9519	1.0568	0.7275	α-Tocopherol	Lipids
432.105098	432.105133	$C_{21}H_{20}O_{10}$	-0.081	$[M + H]_{+}$, $M_{+\bullet}$*	0.0356	0.0622	0.0812	8.3353	0.0094	0.0456	0.0102	1.9340	Kaempferol-3-O-rhamnoside	Flavonoid
434.084363	434.084403	$C_{20}H_{18}O_{11}$	-0.092	$[M + H]_{+}$	N.A	N.A	0.0060	N.A	N.A	N.A	N.A	N.A	Quercetin 3-O-arabinoside	Flavonol
437.144223	437.144207	$C_{21}H_{24}O_{10}$	0.036	$[M + H]_{+}$*, $M_{+\bullet}$	0.0055	N.A	0.0072	N.A	N.A	N.A	N.A	N.A	Phloridzin	Phenolics
439.393443	439.393466	$C_{31}H_{50}O$	-0.073	$[M + H]_{+}$*, $M_{+\bullet}$	0.0000	0.0033	0.0000	0.0056	0.0136	0.0284	0.0088	0.0207	24-Methylenecycloartan-3-one	Sterol
441.409093	441.409057	$C_{31}H_{52}O$	0.082	$[M + H]_{+}$*, $M_{+\bullet}$	N.A	N.A	N.A	0.0095	0.0022	0.0141	N.A	0.0097	24-Methylenecycloartanol	Sterol
443.097273	443.097253	$C_{22}H_{18}O_{10}$	0.047	$[M + H]^{+}$*, $M^{+\bullet}$	N.A	N.A	N.A	0.0338	0.0034	0.0060	N.A	0.0138	Epicatechin-3-gallate	Flavan-3-ol
449.107838	449.107882	$C_{21}H_{20}O_{11}$	-0.097	$[M + H]^{+}$*, $M^{+\bullet}$	0.0240	0.0090	0.0700	N.A	0.0534	0.1067	N.A	0.0938	Astragalin	Flavonoid glucoside
453.139138	453.139174	$C_{21}H_{24}O_{11}$	-0.080	$[M + H]^{+}$	N.A	N.A	0.0153	N.A	N.A	N.A	N.A	N.A	Catechin-3-glucoside	Flavonoid glucoside
461.107838	461.107874	$C_{22}H_{20}O_{11}$	-0.077	$[M + H]^{+}$*, $M^{+\bullet}$	N.A	0.0035	0.0061	0.5062	N.A	N.A	N.A	N.A	Apigenin 7-O-glucuronide methyl ester	Flavonoid glucoside
463.123488	463.123483	$C_{22}H_{22}O_{11}$	0.011	$[M + H]^{+}$*, $M^{+\bullet}$	0.0177	0.0207	0.0331	0.0212	0.0475	0.0665	0.0110	0.0085	Chrysoeriol 7-O-glucoside	Flavonoid
465.102753	465.102787	$C_{21}H_{20}O_{12}$	-0.074	$[M + H]^{+}$*, $M^{+\bullet}$	0.0096	N.A	0.0357	N.A	0.0335	0.0419	N.A	N.A	Hyperoside	Flavonoid
465.357451	465.357423	$C_{28}H_{48}O_5$	0.060	$[M + H]^{+}$*, $M^{+\bullet}$	N.A	0.0104	N.A	0.0042	0.0176	0.0139	0.0217	0.0237	Castasterone	Sterol
478.183349	478.183401	$C_{24}H_{30}O_{10}$	-0.109	$M^{+\bullet}$*	0.2808	0.3169	0.6092	0.0866	0.0807	0.1324	0.0632	0.0193	Junipercomnoside A	
479.118403	479.118376	$C_{22}H_{22}O_{12}$	0.055	$[M + H]^{+}$*, $M^{+\bullet}$	0.0196	N.A	0.0231	0.0070	0.0506	0.0192	N.A	0.0080	3′-O-methylquercetin 3-glucoside	Phenolic glycoside
479.118403	479.118150	$C_{22}H_{22}O_{12}$	0.527	$[M + H]^{+}$	N.A	0.0030	N.A	N.A	N.A	N.A	N.A	N.A	3-O-methylquercetin 3-glucoside	Phenolic glycoside
481.352366	481.352311	$C_{28}H_{48}O_6$	0.114	$[M + H]^{+}$, $M^{+\bullet}$*	N.A	0.0045	N.A	N.A	N.A	N.A	N.A	N.A	Brassinolide	
491.118403	491.118407	$C_{23}H_{22}O_{12}$	-0.010	$[M + H]^{+}$	0.0103	N.A	N.A	N.A	0.0178	N.A	N.A	N.A	Kaempferol 3-(6''-acetylglucoside)	Flavonoid glucoside
495.113317	495.113276	$C_{22}H_{22}O_{13}$	0.084	$[M + H]^{+}$	N.A	N.A	0.0144	N.A	N.A	N.A	N.A	N.A	Laricitrin 3-glucoside	Flavonoid glucoside
506.214649	506.214640	$C_{26}H_{34}O_{10}$	0.016	$[M + H]^{+}$	0.2729	0.4089	0.5120	0.0102	0.0514	0.1578	0.0787	N.A	Icariside E4	
507.113317	507.113332	$C_{23}H_{22}O_{13}$	-0.030	$[M + H]^{+}$	0.0137	N.A	0.0051	N.A	0.0417	N.A	N.A	N.A	Quercetin 3-(6''-acetylglucoside)	Flavonoid glucoside
507.149703	507.149680	$C_{24}H_{26}O_{12}$	0.044	$[M + H]^{+}$*, $M^{+\bullet}$	0.0098	0.0345	N.A	N.A	0.0126	0.1187	N.A	N.A	Junipegenin B 7-O-glucoside	Flavonoid glucoside

508.193913	508.193894	$C_{25}H_{32}O_{11}$	0.037	$M^{+\bullet}$	0.1019	0.8397	1.5628	0.0717	0.0228	0.3341	0.1169	0.0162	Gibberellin 2-O-beta-D-glucoside	
509.128967	509.128861	$C_{23}H_{24}O_{13}$	0.208	$[M + H]^{+}$	N.A	N.A	0.0102	N.A	N.A	N.A	N.A	N.A	Syringetin 3-glucoside	Flavonoid glucoside
522.209563	522.209520	$C_{26}H_{34}O_{11}$	0.084	$M^{+\bullet}$*	N.A	0.0728	N.A	0.0241	N.A	N.A	N.A	N.A	lariciresinol glycoside	Lignan
526.183349	526.183339	$C_{28}H_{30}O_{10}$	0.018	$M^{+\bullet}$*	0.0063	0.0102	0.0136	N.A	N.A	0.0092	N.A	N.A	Pilosanol B	
534.209563	534.209472	$C_{27}H_{34}O_{11}$	0.172	$[M + H]^{+}$, $M^{+\bullet}$*	N.A	0.0110	0.0498	N.A	N.A	N.A	N.A	N.A	Arctiin	Lignan
538.089448	538.089343	$C_{30}H_{18}O_{10}$	0.195	$M^{+\bullet}$	N.A	N.A	0.0372	N.A	N.A	N.A	N.A	N.A	Sennidin A, Cupressuflavone	Flavonoid
556.230299	556.230244	$C_{30}H_{36}O_{10}$	0.098	$M^{+\bullet}$	0.0057	0.0190	0.0337	0.0036	N.A	0.0220	N.A	N.A	Lariciresinol-sesquilignan	Lignan
559.144617	559.144632	$C_{27}H_{26}O_{13}$	-0.026	$[M + H]^{+}$	N.A	N.A	N.A	0.4405	N.A	N.A	N.A	N.A	Piceatannol 4'-galloylglucoside	Stilbenoid
578.141878	578.141689	$C_{30}H_{26}O_{12}$	0.326	$[M + H]^{+}$*, $M^{+\bullet}$	N.A	N.A	0.0049	N.A	N.A	N.A	N.A	N.A	Procyanidin dimer B1	Tannins
578.163007	578.163024	$C_{27}H_{30}O_{14}$	-0.029	$[M + H]^{+}$, $M^{+\bullet}$*	N.A	N.A	N.A	4.3147	N.A	N.A	N.A	1.2733	Isorhoifolin, Vitexin 2''-O-rhamnoside	Flavonoid glucoside

*Major ion
